# Di-μ-acetato-bis­(dimethyl­formamide)­penta­kis­(μ-*N*,2-dioxidobenzene-1-car­boximidato)tetra­kis­(1-ethyl­imidazole)­penta­manganese(III)­manganese(II)–diethyl ether–dimethyl­foramide–methanol–water (1/1/1/1/0.12)

**DOI:** 10.1107/S160053681102602X

**Published:** 2011-07-09

**Authors:** Benjamin R. Tigyer, Matthias Zeller, Curtis M. Zaleski

**Affiliations:** a Department of Chemistry, Shippensburg University, 1871 Old Main Dr., Shippensburg, PA 17257, USA; b Department of Chemistry, Youngstown State University, 1 University Plaza, Youngstown, OH 44555, USA

## Abstract

The title compound [Mn_6_(C_7_H_4_NO_3_)_5_(CH_3_CO_2_)_2_(C_5_H_8_N_2_)_4_(C_3_H_7_NO)_2_]·(C_2_H_5_)_2_O·C_3_H_7_NO·CH_3_OH·0.12H_2_O, abbreviated as Mn^II^(OAc)_2_[15-MC_MnIII(N)shi_-5](EtIm)4(DMF)2·diethyl ether·DMF·MeOH·0.12H_2_O (where ^−^OAc is acetate, MC is metallacrown, shi^3−^ is salicylhydroximate, EtIM is *n*-ethylimidazole, DMF is *N*,*N*-dimethylformamide, and MeOH is methanol) contains five Mn^III^ ions as members of the metallacrown ring and an Mn^II^ ion bound in the central cavity. The central Mn^II^ ion is seven-coordinate with a distorted face-capped trigonal–prismatic geometry. The five Mn^III^ ions of the metallacrown ring are six-coordinate with distorted octa­hedral geometries. The configuration of the Mn^III^ ions about the metallacrown ring follow a ΔΛΔ*PP* pattern, with *P* representing planar. The four 1-ethyl­imidazole ligands are bound to four different Mn^III^ ions. A diethyl ether solvent mol­ecule was found to be disordered over two mutually exclusive sites with an occupancy ratio of 0.568 (7):0.432 (7). A methanol solvent mol­ecule was found to be disordered over two mutually exclusive sites by being hydrogen bonded either to a dimethyl­formamide solvent mol­ecule (major occupancy component) or to an O atom of the main mol­ecule (minor occupancy component). The occupancy ratio refined to 0.678 (11):0.322 (11). Associated with the minor component is a partially occupied water mol­ecule [total occupancy 0.124 (15)].

## Related literature

For a general review of metallacrowns, see: Mezei *et al.* (2007 ▶); Pecoraro *et al.* (1997 ▶). For related Mn^II^[15-MC_MnIIIN(shi)_-5)] structures, see: Kessissoglou *et al.* (1994 ▶); Dendrinou-Samara *et al.* (2002 ▶, 2005 ▶); Emerich *et al.* (2010 ▶). For an explanation of how to calculate the *s/h* ratio, see: Stiefel & Brown (1972 ▶). For the preparation of {Mn^II^(OAc)_2_[12-MC_MnIIIN(shi)_-4](DMF)_6_}·2DMF, see: Lah & Pecoraro (1989 ▶).
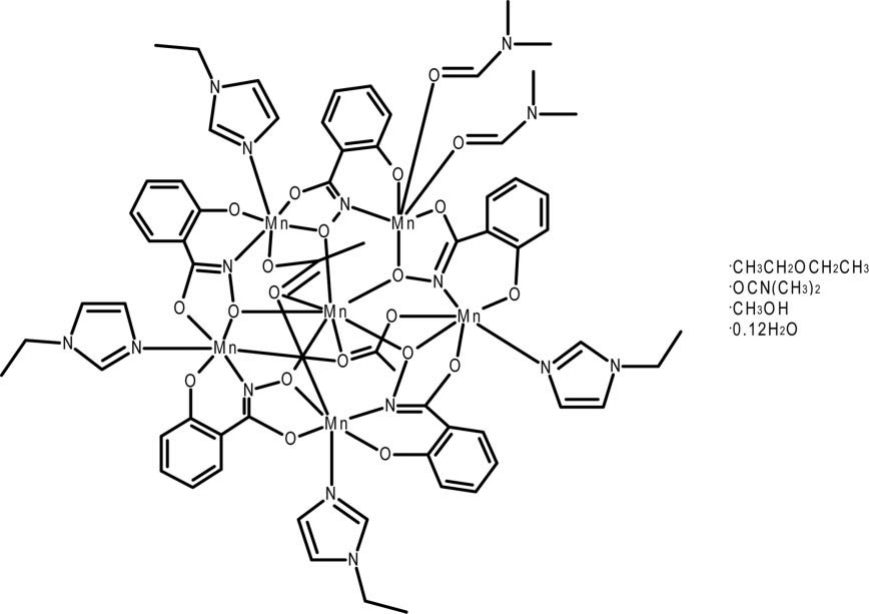


## Experimental

### 

#### Crystal data

[Mn_6_(C_7_H_4_NO_3_)_5_(C_2_H_3_O_2_)_2_(C_5_H_8_N_2_)_4_(C_3_H_7_NO)_2_]·C_4_H_10_O·C_3_H_7_NO·CH_4_O·0.12H_2_O
*M* *_r_* = 1910.51
Triclinic, 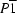

*a* = 12.604 (2) Å
*b* = 17.188 (3) Å
*c* = 20.990 (4) Å
α = 103.564 (3)°
β = 97.322 (3)°
γ = 107.658 (3)°
*V* = 4114.2 (13) Å^3^
*Z* = 2
Mo *K*α radiation
μ = 0.98 mm^−1^
*T* = 100 K
0.35 × 0.29 × 0.13 mm

#### Data collection

Bruker SMART APEX CCD diffractometer
Absorption correction: multi-scan (*SADABS*; Bruker, 2009 ▶) *T*_min_ = 0.563, *T*_max_ = 0.746
49270 measured reflections
18692 independent reflections
14786 reflections with *I* > 2σ(*I*)
*R*_int_ = 0.046

#### Refinement

*R*[*F* ^2^ > 2σ(*F* ^2^)] = 0.059
*wR*(*F* ^2^) = 0.148
*S* = 1.07
18692 reflections
1143 parameters
53 restraints
H atoms treated by a mixture of independent and constrained refinement
Δρ_max_ = 1.53 e Å^−3^
Δρ_min_ = −0.76 e Å^−3^

### 

Data collection: *APEX2* (Bruker, 2009 ▶); cell refinement: *SAINT* (Bruker, 2009 ▶); data reduction: *SAINT*; program(s) used to solve structure: *SHELXTL* (Sheldrick, 2008 ▶); program(s) used to refine structure: *SHELXTL*; molecular graphics: *SHELXTL*, *Mercury* (Macrae *et al.*, 2006 ▶) and *ORTEP-3* (Farrugia, 1997 ▶); software used to prepare material for publication: *SHELXTL*.

## Supplementary Material

Crystal structure: contains datablock(s) I, global. DOI: 10.1107/S160053681102602X/jj2091sup1.cif

Structure factors: contains datablock(s) I. DOI: 10.1107/S160053681102602X/jj2091Isup2.hkl

Additional supplementary materials:  crystallographic information; 3D view; checkCIF report

## Figures and Tables

**Table 1:** Hydrogen-bond geometry (Å, °)

*D*—H⋯*A*	*D*—H	H⋯*A*	*D*⋯*A*	*D*—H⋯*A*
O24—H24*A*⋯O22	0.84	2.09	2.876 (7)	156
O24*B*—H24*B*⋯O19	0.84	2.15	2.98 (2)	170
O25—H25*A*⋯O24*B*	0.84 (2)	2.35 (2)	3.05 (4)	140 (6)
O25—H25*B*⋯O22	0.84 (2)	1.99 (2)	2.776 (17)	156 (4)

**Fig. 1.**
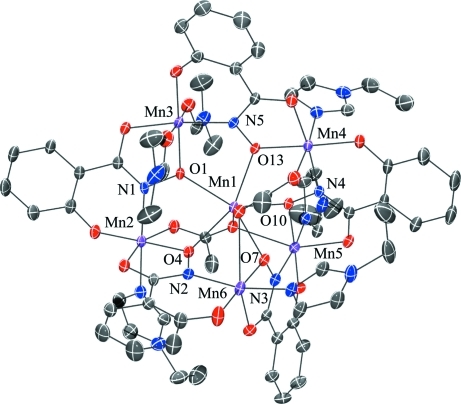
Single-crystal X-ray structure of MnII(OAc)2[15-MCMnIIIN(shi)-5](EtIm)4(DMF)2.Diethyl ether.DMF.MeOH.0.14H2O (1). The thermal ellipsoid plot of 1 is at a 50% probability level. All atoms composing the MC ring are labeled. Hydrogen atoms and the lattice solvent molecules have been omitted for clarity. Color scheme for all figures: purple - MnII and MnIII, red - oxygen, blue - nitrogen, and gray - carbon.

**Fig. 2.**
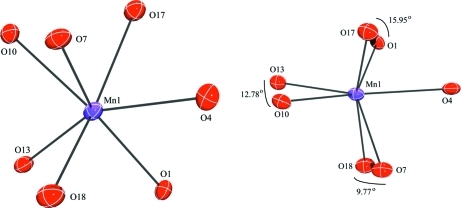
Side (left) and top (right) views of the first coordination sphere about Mn1 (2+ oxidation state) of 1 with face-capped trigonal prismatic geometry. The thermal ellipsoid plots are at a 50% probability level.

**Fig. 3.**
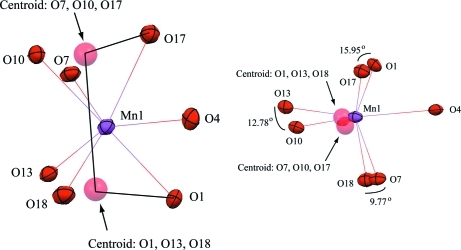
Side (left) and top (right) views of the first coordination sphere about Mn1 of 1 indicating how the azimuthal angle (Φ) was defined and calculated with the program Mercury (Macrae et al., 2006). The thermal ellipsoid plots are at a 50% probability level.

**Fig. 4.**
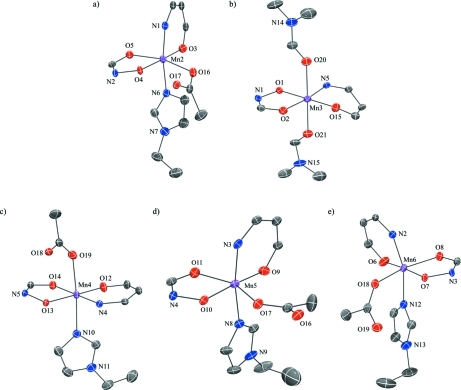
First coordination sphere about each MnIII ion of 1. a) Mn2 with Δ configuration b) Mn3 with planar configuration c) Mn4 with planar configuration d) Mn5 with Δ configuration and e) Mn6 with Λ configuration. The thermal ellipsoid plots are at a 50% probability level. Hydrogen atoms have been omitted for clarity.

**Fig. 5.**
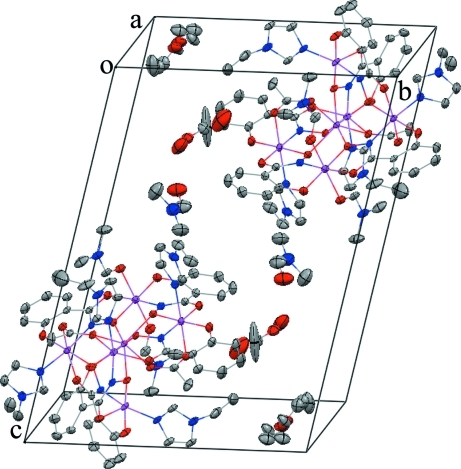
Packing diagram of 1 along the a axis. The thermal ellipsoid plot is at a 50% probability level. Hydrogen atoms have been omitted for clarity.

## supplementary crystallographic information

### Comment

Metallacrowns (MCs) are a versatile class of inorganic molecules that serve both as structural and functional analogues to crown ethers (Mezei *et al.*, 2007; Pecoraro *et al.*, 1997). In addition to mimicking crown ethers, MCs have other potential uses such as single-molecule magnets, MRI contrast agents, and catalysts (Mezei *et al.*, 2007). The naming scheme for MCs is similar to that of crown ethers. For example, 15-MC-5 indicates that the ring size of the MC consists of 15 atoms and that there are 5 oxygen atoms capable of binding a metal ion in the central cavity. A complete naming scheme can be found in the review by Pecoraro *et al.* (1997). Manganese-based 15-MC-5 structures were the first 15-MC-5 compounds reported (Kessissoglou *et al.*, 1994), and some of these molecules have shown antibacterial activity (Dendrinou-Samara *et al.*, 2002; Dendrinou-Samara *et al.*, 2005).

Herein we report the synthesis, IR data, and the single-crystal X-ray structure of the title compound,[Mn_6_(C_7_H_4_NO_3_)_5_(C_5_N_2_H_8_)_4_(C_3_H_7_NO)_2_(C_2_H_3_O_2_)_2_].(C_2_H_5_)_2_O.C_3_H_7_NO.CH_3_OH.0.12H_2_O, 1, abbreviated as Mn^II^(OAc)_2_[15-MC_Mn^III^N(shi)_-5](EtIm)_4_(DMF)_2_.Diethyl ether.DMF.MeOH.0.12H_2_O (where ^-^OAc is acetate, MC is metallacrown, shi^3-^ is salicylhydroximate, EtIM is *n*-ethylimidazole, DMF is *N*,*N*-dimethylformamide, and MeOH is methanol). The single-crystal X-ray structure of a related molecule, Mn^II^(OAc)_2_[15-MC_Mn^III^N(shi)_-5](Im)_3_(EtOH)_3_ (where Im is imidazole and EtOH is ethanol), has previously been reported by Emerich *et al.* (2010).

Compound 1 is a non-planar molecule, which is typical of Mn^II^[15-MC_Mn^III^_-5] structures (Fig. 1–5; Farrugia, 1997). The structure consists of a [Mn^III^—N—O]_5_ repeat unit around the MC ring, and the MC binds a Mn^II^ in the central cavity (Fig. 1). The positive charge of the five Mn^III^ ions and the one Mn^II^ ion are counterbalanced by the five shi^3-^ ligands and two acetate anions. Mn1 is located in the central cavity and is seven-coordinate with distorted face-capped trigonal prismatic geometry (Fig. 2). The geometry assignment is based on the calculated azimuthal angle (Φ). In an ideal face-capped octahedron Φ = 60°, while in an ideal face-capped trigonal prism Φ = 0°. To calculate the Φ angle, the centers of opposite triangular faces made by the donor oxygen atoms (O1, O13, and O18; O7, O10, and O17) were defined using the program Mercury (Macrae *et al.*, 2006), and then twist angles between atoms on opposite faces through the centroids were calculated (Fig. 3). The calculated Φ angles of 9.77°, 12.78°, and 15.95° indicate the geometry approaches more that of face-capped trigonal prismatic. Another parameter that indicates the geometry is the *s/h* ratio (Stiefel and Brown, 1972). In an ideal octahedron the *s/h* ratio is 1.22, while in an ideal trigonal prism the *s/h* ratio is 1.00. Defining the distance between the centroids in Fig. 3 as *h*, the average *s/h* ratio for 1 is 1.00 ± 0.10. Thus both the Φ angle and the *s/h* ratio support a distorted face-capped trigonal prismatic geometry. The assignment of 2+ for Mn1 is supported by an average bond distance of 2.24 Å. The ring Mn (2–6) are assigned a 3+ oxidation state. This is supported by the average bond distance, and the observation of a Jahn-Teller axis for each ring Mn, which is typical for a high spin *d*^4^ cation. The average bond distances for Mn2, Mn3, Mn4, Mn5, and Mn6 are 2.03 Å, 2.03 Å, 2.03 Å, 2.05 Å, and 2.09 Å, respectively. Each Mn^III^ is six-coordinate with a distorted octahedral geometry (Fig. 4). It should be noted that Mn6 has one long interaction with an acetate oxygen atom (Mn6—O18, 2.684 (3) Å) that is needed to complete the octahedral geometry. The coordination about the Mn^III^ ions can also be described by their configurations. Mn2 has a propeller configuration with Δ absolute stereochemistry. Mn3 and Mn4 do not adopt a propeller configuration, but instead two shi^3-^ ligands are *trans* to each other. Thus, Mn3 and Mn4 adopt a planar (P) configuration. Mn5 has a propeller configuration with Δ absolute stereochemistry, and Mn6 has a propeller configuration with Λ absolute stereochemistry. Starting with Mn5, the configuration pattern for the Mn^III^ ions about the MC ring is ΔΛΔPP. In addition, Mn2, Mn4, Mn5, and Mn6 bind *n*-ethylimidazole ligands, which are directed to the periphery of the molecule. Lastly, a disordered diethyl ether molecule, a disordered methanol molecule, a dimethylformamide molecule, and a partially occupied water molecule (0.124 (15)) are located in the lattice.

### Experimental

Manganese(II) acetate tetrahydrate (99+%) and *N,N*-dimethylformamide (ACS grade) were purchased from Acros Organics, salicylhydroxamic acid (H_3_shi, 99%) was purchased from Alfa Aesar, *n*-ethylimidazole (98%) was purchased from TCI America, methanol (HPLC grade) was purchased from Fisher Scientific, and absolute diethyl ether was purchased from EMD Chemicals. All reagents were used as received and without further purification.

The compound {Mn^II^(OAc)_2_[12-MC_Mn^III^N(shi)_-4](DMF)_6_}.2DMF was prepared as previously reported (Lah & Pecoraro, 1989), and dark brown crystals were isolated and dried. Then 0.1 mmol of the {Mn^II^(OAc)_2_[12-MC_Mn^III^N(shi)_-4](DMF)_6_}.2DMF compound was dissolved in 20 ml of a 50:50 mixture of methanol and DMF. The resulting mixture was a dark brown color. Then 25 µ*L* of *n*-ethylimidazole was added to this solution, and the solution was stirred for 5 minutes. The resulting dark brown solution was filtered and no precipitate was recovered. Diffusion of diethyl ether into the filtrate at approximately 277 K (4 °C) resulted in dark brown cube-like crystals after 9 days. The percent yield was 22% based on the {Mn^II^(OAc)_2_[12-MC_Mn^III^N(shi)_-4](DMF)_6_}.2DMF starting material. Elemental analysis for the dried material (accounting for the loss of the diethyl ether lattice solvent) C_69_H_83.24_Mn_6_N_16_O_23.12_ [FW = 1836.28 g/mol] found % (calculated); C 45.59 (45.13); H 4.44 (4.57); N 12.37 (12.20).

### Refinement

A solvent diethyl ether molecule was found to be disordered over two mutually exclusive sites. The molecules were restrained to have similar geometries and equivalent atoms were constrained to have identical ADPs. The occupancy ratio refined to 0.568 (7) to 0.432 (7). A solvent methanol molecule was found to be disordered over two mutually exclusive sites by being hydrogen bonded either to a solvent DMF molecule (major moiety) or to an oxygen atom of the main molecule (minor moiety). The two molecules were restrained to have similar C—O distances. The occupancy ratio refined to 0.678 (11) to 0.322 (11). Associated with the minor moiety is a partially occupied water molecule (total occupancy: 0.124 (15). This position of the water oxygen atom and of its H atoms was restrained based on hydrogen bonding considerations. It was also restrained to be approximately isotropic. The carbon atoms of one ethyl group (C48, C49) showed signs of unresolved disorder and were restrained to be approximately isotropic.

Hydrogen atoms were placed in calculated positions with C—H = 0.95 (aromatic), 0.98 (methyl) and 0.99 Å (methylene) and were refined with Uĩso~(H) = 1.5 *U*_eq_(C) for methyl H atoms and 1.2 *U*_eq_(C) for methylene and aromatic moieties.

### Crystal data

**Table:**

[Mn_6_(C_7_H_4_NO_3_)_5_(C_2_H_3_O_2_)_2_(C_5_H_8_N_2_)_4_(C_3_H_7_NO)_2_]·C_4_H_10_O·C_3_H_7_NO·CH_4_O·0.12H_2_O	*Z* = 2
*M**_r_* = 1910.51	*F*(000) = 1972.1
Triclinic, *P*1	*D*_x_ = 1.542 Mg m^−^^3^
Hall symbol: -P 1	Mo *K*α radiation, λ = 0.71073 Å
*a* = 12.604 (2) Å	Cell parameters from 1712 reflections
*b* = 17.188 (3) Å	θ = 2.7–30.2°
*c* = 20.990 (4) Å	µ = 0.98 mm^−^^1^
α = 103.564 (3)°	*T* = 100 K
β = 97.322 (3)°	Plate, black
γ = 107.658 (3)°	0.35 × 0.29 × 0.13 mm
*V* = 4114.2 (13) Å^3^	

### Data collection

**Table:**

Bruker SMART APEX CCD diffractometer	18692 independent reflections
Radiation source: fine-focus sealed tube	14786 reflections with *I* > 2σ(*I*)
graphite	*R*_int_ = 0.046
ω scans	θ_max_ = 27.5°, θ_min_ = 1.7°
Absorption correction: multi-scan (*SADABS*; Bruker, 2009)	*h* = −16→16
*T*_min_ = 0.563, *T*_max_ = 0.746	*k* = −22→22
49270 measured reflections	*l* = −27→27

### Refinement

**Table:**

Refinement on *F*^2^	Primary atom site location: structure-invariant direct methods
Least-squares matrix: full	Secondary atom site location: difference Fourier map
*R*[*F*^2^ > 2σ(*F*^2^)] = 0.059	Hydrogen site location: inferred from neighbouring sites
*wR*(*F*^2^) = 0.148	H atoms treated by a mixture of independent and constrained refinement
*S* = 1.07	*w* = 1/[σ^2^(*F*_o_^2^) + (0.0581*P*)^2^ + 8.5964*P*] where *P* = (*F*_o_^2^ + 2*F*_c_^2^)/3
18692 reflections	(Δ/σ)_max_ = 0.002
1143 parameters	Δρ_max_ = 1.53 e Å^−^^3^
53 restraints	Δρ_min_ = −0.76 e Å^−^^3^
5 constraints	

### Special details

**Table:**

Experimental. FT–IR bands (KBr pellet, cm^-1^): 1655(*w*), 1598(*m*), 1572(*s*), 1500(*s*), 1438(*m*), 1389(*m*), 1320(*m*), 1259(*m*), 1100(*m*), 1032(*w*), 927(*m*), 866(*m*), 755(*m*), 681(*m*), 651(*m*), 603(*m*), and 470(*m*).
Geometry. All e.s.d.'s (except the e.s.d. in the dihedral angle between two l.s. planes) are estimated using the full covariance matrix. The cell e.s.d.'s are taken into account individually in the estimation of e.s.d.'s in distances, angles and torsion angles; correlations between e.s.d.'s in cell parameters are only used when they are defined by crystal symmetry. An approximate (isotropic) treatment of cell e.s.d.'s is used for estimating e.s.d.'s involving l.s. planes.
Refinement. Refinement of *F*^2^ against ALL reflections. The weighted *R*-factor *wR* and goodness of fit *S* are based on *F*^2^, conventional *R*-factors *R* are based on *F*, with *F* set to zero for negative *F*^2^. The threshold expression of *F*^2^ > σ(*F*^2^) is used only for calculating *R*-factors(gt) *etc*. and is not relevant to the choice of reflections for refinement. *R*-factors based on *F*^2^ are statistically about twice as large as those based on *F*, and *R*- factors based on ALL data will be even larger.

### Fractional atomic coordinates and isotropic or equivalent isotropic displacement parameters (Å^2^)

**Table:**

	*x*	*y*	*z*	*U*_iso_*/*U*_eq_	Occ. (<1)
C1	0.7350 (3)	0.9084 (2)	0.31868 (17)	0.0173 (7)	
C2	0.7214 (3)	0.9888 (2)	0.31474 (17)	0.0182 (7)	
C3	0.6292 (3)	1.0079 (3)	0.33682 (19)	0.0227 (8)	
H3	0.5784	0.9684	0.3537	0.027*	
C4	0.6105 (4)	1.0824 (3)	0.3347 (2)	0.0282 (9)	
H4	0.5471	1.0939	0.3494	0.034*	
C5	0.6855 (4)	1.1403 (3)	0.3108 (2)	0.0342 (10)	
H5	0.6734	1.1918	0.3092	0.041*	
C6	0.7777 (4)	1.1239 (3)	0.2893 (2)	0.0299 (9)	
H6	0.8289	1.1648	0.2738	0.036*	
C7	0.7970 (3)	1.0476 (2)	0.28988 (18)	0.0211 (8)	
C8	0.8083 (3)	0.8217 (2)	0.10425 (18)	0.0165 (7)	
C9	0.7472 (3)	0.7836 (2)	0.03382 (18)	0.0183 (7)	
C10	0.6736 (3)	0.8210 (3)	0.0078 (2)	0.0237 (8)	
H10	0.6659	0.8702	0.0360	0.028*	
C11	0.6124 (4)	0.7884 (3)	−0.0575 (2)	0.0318 (10)	
H11	0.5624	0.8144	−0.0738	0.038*	
C12	0.6242 (4)	0.7168 (3)	−0.0995 (2)	0.0406 (12)	
H12	0.5832	0.6945	−0.1448	0.049*	
C13	0.6953 (4)	0.6788 (3)	−0.0752 (2)	0.0381 (11)	
H13	0.7027	0.6299	−0.1040	0.046*	
C14	0.7572 (3)	0.7108 (3)	−0.00860 (19)	0.0251 (8)	
C15	1.1539 (3)	0.7569 (2)	0.13292 (17)	0.0166 (7)	
C16	1.2767 (3)	0.7965 (2)	0.13606 (18)	0.0186 (7)	
C17	1.3110 (3)	0.8163 (3)	0.07890 (19)	0.0233 (8)	
H17	1.2548	0.8067	0.0404	0.028*	
C18	1.4246 (4)	0.8494 (3)	0.0778 (2)	0.0297 (9)	
H18	1.4465	0.8619	0.0386	0.036*	
C19	1.5073 (3)	0.8644 (3)	0.1342 (2)	0.0295 (9)	
H19	1.5858	0.8871	0.1336	0.035*	
C20	1.4755 (3)	0.8463 (3)	0.19097 (19)	0.0245 (8)	
H20	1.5327	0.8571	0.2293	0.029*	
C21	1.3603 (3)	0.8121 (2)	0.19326 (18)	0.0185 (7)	
C22	1.1045 (3)	0.5681 (2)	0.26788 (17)	0.0181 (7)	
C23	1.0764 (3)	0.4758 (2)	0.25878 (19)	0.0210 (8)	
C24	1.1490 (4)	0.4368 (3)	0.2295 (2)	0.0281 (9)	
H24	1.2115	0.4701	0.2153	0.034*	
C25	1.1311 (4)	0.3516 (3)	0.2210 (3)	0.0384 (11)	
H25	1.1800	0.3259	0.2006	0.046*	
C26	1.0410 (5)	0.3038 (3)	0.2427 (3)	0.0454 (13)	
H26	1.0286	0.2451	0.2375	0.055*	
C27	0.9687 (4)	0.3406 (3)	0.2720 (3)	0.0388 (11)	
H27	0.9078	0.3069	0.2870	0.047*	
C28	0.9839 (3)	0.4266 (2)	0.2798 (2)	0.0229 (8)	
C29	0.6962 (3)	0.5839 (2)	0.34485 (18)	0.0170 (7)	
C30	0.5775 (3)	0.5644 (2)	0.35268 (18)	0.0193 (7)	
C31	0.5095 (3)	0.4788 (3)	0.3384 (2)	0.0281 (9)	
H31	0.5411	0.4357	0.3243	0.034*	
C32	0.3977 (4)	0.4559 (3)	0.3444 (3)	0.0361 (10)	
H32	0.3518	0.3974	0.3334	0.043*	
C33	0.3521 (3)	0.5193 (3)	0.3668 (2)	0.0313 (9)	
H33	0.2751	0.5040	0.3715	0.038*	
C34	0.4183 (3)	0.6037 (3)	0.3820 (2)	0.0257 (8)	
H34	0.3863	0.6461	0.3978	0.031*	
C35	0.5311 (3)	0.6289 (2)	0.37494 (18)	0.0204 (7)	
C36	1.1309 (3)	0.9193 (2)	0.31077 (17)	0.0188 (7)	
C37	1.2511 (4)	0.9685 (3)	0.3086 (3)	0.0376 (11)	
H37A	1.3047	0.9505	0.3346	0.056*	
H37B	1.2669	1.0296	0.3278	0.056*	
H37C	1.2599	0.9572	0.2619	0.056*	
C38	0.7527 (3)	0.5603 (2)	0.17671 (18)	0.0205 (8)	
C39	0.6527 (4)	0.5058 (3)	0.1190 (2)	0.0378 (11)	
H39A	0.6614	0.4508	0.1002	0.057*	
H39B	0.6503	0.5349	0.0842	0.057*	
H39C	0.5817	0.4963	0.1354	0.057*	
C40	1.0971 (3)	0.9664 (3)	0.15302 (19)	0.0240 (8)	
H40	1.0773	0.9074	0.1309	0.029*	
C41	1.0974 (4)	1.0860 (3)	0.2155 (2)	0.0304 (9)	
H41	1.0765	1.1264	0.2458	0.036*	
C42	1.1876 (4)	1.1041 (3)	0.1868 (2)	0.0350 (10)	
H42	1.2416	1.1591	0.1929	0.042*	
C43	1.2720 (4)	1.0180 (3)	0.1069 (3)	0.0408 (11)	
H43A	1.2749	1.0529	0.0753	0.049*	
H43B	1.2486	0.9575	0.0802	0.049*	
C44	1.3887 (4)	1.0444 (4)	0.1505 (3)	0.0517 (14)	
H44A	1.4134	1.1048	0.1757	0.078*	
H44B	1.4427	1.0355	0.1223	0.078*	
H44C	1.3861	1.0100	0.1819	0.078*	
C45	1.4080 (3)	0.8109 (2)	0.38991 (18)	0.0209 (8)	
H45	1.4599	0.8226	0.3614	0.025*	
C46	1.2529 (4)	0.7707 (3)	0.42595 (19)	0.0269 (9)	
H46	1.1744	0.7484	0.4267	0.032*	
C47	1.3402 (4)	0.7998 (3)	0.4797 (2)	0.0329 (10)	
H47	1.3347	0.8025	0.5248	0.040*	
C48	1.5551 (4)	0.8619 (4)	0.4977 (3)	0.0527 (15)	
H48A	1.5576	0.8415	0.5380	0.063*	
H48B	1.6070	0.8424	0.4715	0.063*	
C49	1.5945 (7)	0.9545 (6)	0.5184 (4)	0.107 (3)	
H49A	1.6087	0.9754	0.4795	0.161*	
H49B	1.6652	0.9768	0.5527	0.161*	
H49C	1.5362	0.9739	0.5368	0.161*	
C50	1.0591 (3)	0.5772 (3)	0.4538 (2)	0.0242 (8)	
H50	1.0772	0.5308	0.4291	0.029*	
C51	0.9833 (4)	0.6726 (3)	0.4804 (2)	0.0315 (9)	
H51	0.9368	0.7066	0.4775	0.038*	
C52	1.0597 (4)	0.6823 (3)	0.5357 (2)	0.0363 (10)	
H52	1.0764	0.7233	0.5782	0.044*	
C53	1.1973 (4)	0.6080 (3)	0.5631 (2)	0.0383 (11)	
H53A	1.1649	0.5880	0.5993	0.046*	
H53B	1.2600	0.6633	0.5842	0.046*	
C54	1.2436 (5)	0.5459 (4)	0.5275 (3)	0.0487 (13)	
H54A	1.1823	0.4906	0.5078	0.073*	
H54B	1.3029	0.5400	0.5591	0.073*	
H54C	1.2764	0.5657	0.4918	0.073*	
C55	0.9302 (3)	0.5140 (2)	0.09391 (19)	0.0238 (8)	
H55	0.9678	0.5353	0.1399	0.029*	
C56	0.8500 (3)	0.5057 (3)	−0.00588 (19)	0.0258 (9)	
H56	0.8201	0.5210	−0.0431	0.031*	
C57	0.8496 (4)	0.4272 (3)	−0.00621 (19)	0.0284 (9)	
H57	0.8201	0.3773	−0.0435	0.034*	
C58	0.9067 (5)	0.3603 (3)	0.0823 (2)	0.0461 (13)	
H58A	0.9742	0.3804	0.1195	0.055*	
H58B	0.9165	0.3164	0.0458	0.055*	
C59	0.8014 (6)	0.3212 (4)	0.1065 (3)	0.0619 (18)	
H59A	0.7933	0.3640	0.1438	0.093*	
H59B	0.8080	0.2728	0.1217	0.093*	
H59C	0.7344	0.3014	0.0698	0.093*	
C60	0.9350 (3)	0.8617 (3)	0.44657 (19)	0.0253 (8)	
H60	0.9580	0.8621	0.4052	0.030*	
C61	0.9857 (5)	0.9008 (4)	0.5669 (2)	0.0543 (15)	
H61A	1.0111	0.9598	0.5954	0.081*	
H61B	1.0236	0.8684	0.5883	0.081*	
H61C	0.9030	0.8748	0.5609	0.081*	
C62	1.1338 (4)	0.9394 (4)	0.5011 (3)	0.0477 (14)	
H62A	1.1409	0.9352	0.4546	0.072*	
H62B	1.1796	0.9101	0.5203	0.072*	
H62C	1.1611	0.9996	0.5274	0.072*	
C66	0.4691 (5)	0.3119 (5)	0.4271 (3)	0.0678 (18)	
H66	0.5159	0.3599	0.4633	0.081*	
C67	0.2814 (6)	0.2221 (4)	0.3633 (4)	0.079 (2)	
H67A	0.2277	0.2423	0.3398	0.119*	
H67B	0.2390	0.1750	0.3792	0.119*	
H67C	0.3262	0.2021	0.3325	0.119*	
C68	0.3059 (6)	0.3397 (5)	0.4664 (3)	0.0664 (18)	
H68A	0.3655	0.3916	0.4960	0.100*	
H68B	0.2689	0.3046	0.4934	0.100*	
H68C	0.2492	0.3552	0.4407	0.100*	
Mn1	0.93174 (4)	0.73894 (3)	0.26039 (3)	0.01331 (11)	
Mn2	0.91283 (5)	0.93489 (3)	0.23478 (3)	0.01607 (12)	
Mn3	0.70262 (4)	0.75746 (3)	0.34634 (3)	0.01606 (12)	
Mn4	0.89055 (5)	0.56145 (3)	0.31923 (3)	0.01619 (12)	
Mn5	1.20451 (4)	0.74267 (3)	0.27382 (3)	0.01525 (12)	
Mn6	0.91421 (5)	0.68460 (3)	0.09187 (3)	0.01735 (12)	
N1	0.8087 (3)	0.88169 (18)	0.28918 (14)	0.0157 (6)	
N2	0.8782 (2)	0.78744 (19)	0.12949 (14)	0.0155 (6)	
N3	1.1224 (2)	0.72978 (19)	0.18318 (15)	0.0159 (6)	
N4	1.0339 (2)	0.60427 (18)	0.29254 (14)	0.0154 (6)	
N5	0.7520 (2)	0.65889 (19)	0.33859 (14)	0.0156 (6)	
N6	1.0400 (3)	0.9991 (2)	0.19402 (16)	0.0207 (6)	
N7	1.1873 (3)	1.0286 (2)	0.14710 (18)	0.0295 (8)	
N8	1.2962 (3)	0.7787 (2)	0.36986 (15)	0.0185 (6)	
N9	1.4381 (3)	0.8247 (2)	0.45671 (16)	0.0278 (8)	
N10	0.9831 (3)	0.6061 (2)	0.42876 (16)	0.0215 (7)	
N11	1.1087 (3)	0.6207 (2)	0.51842 (17)	0.0272 (7)	
N12	0.9013 (3)	0.5605 (2)	0.05783 (15)	0.0193 (6)	
N13	0.8998 (3)	0.4328 (2)	0.05724 (17)	0.0285 (8)	
N14	1.0143 (3)	0.8996 (2)	0.50281 (17)	0.0298 (8)	
N16	0.3571 (4)	0.2912 (3)	0.4201 (2)	0.0546 (12)	
O1	0.8154 (2)	0.80558 (15)	0.29986 (12)	0.0158 (5)	
O2	0.6731 (2)	0.86350 (16)	0.35111 (13)	0.0194 (5)	
O3	0.8877 (2)	1.03649 (16)	0.26824 (14)	0.0224 (6)	
O4	0.9324 (2)	0.82945 (15)	0.19743 (12)	0.0168 (5)	
O5	0.7943 (2)	0.88760 (16)	0.14020 (12)	0.0195 (5)	
O6	0.8240 (3)	0.66976 (19)	0.01068 (14)	0.0312 (7)	
O7	1.00397 (19)	0.69177 (15)	0.17379 (12)	0.0158 (5)	
O8	1.0800 (2)	0.74734 (16)	0.08066 (12)	0.0191 (5)	
O9	1.3382 (2)	0.79776 (18)	0.25096 (13)	0.0221 (6)	
O10	1.0677 (2)	0.69178 (15)	0.30147 (12)	0.0157 (5)	
O11	1.1935 (2)	0.61166 (16)	0.25236 (13)	0.0205 (5)	
O12	0.9099 (2)	0.45570 (17)	0.30834 (14)	0.0239 (6)	
O13	0.8652 (2)	0.66919 (15)	0.33249 (12)	0.0153 (5)	
O14	0.7426 (2)	0.52599 (16)	0.34214 (13)	0.0200 (5)	
O15	0.5925 (2)	0.71167 (17)	0.39152 (13)	0.0218 (6)	
O16	1.0602 (2)	0.95691 (16)	0.31717 (13)	0.0211 (5)	
O17	1.1059 (2)	0.84037 (16)	0.30492 (12)	0.0181 (5)	
O18	0.7927 (2)	0.63903 (16)	0.18156 (12)	0.0197 (5)	
O19	0.7904 (2)	0.52364 (17)	0.21420 (13)	0.0247 (6)	
O20	0.8328 (2)	0.82585 (18)	0.44472 (13)	0.0254 (6)	
O21	0.5729 (2)	0.69487 (19)	0.25284 (14)	0.0281 (6)	
C63	0.5769 (3)	0.6919 (3)	0.1935 (2)	0.0270 (9)	
H63	0.6458	0.7221	0.1830	0.032*	
C64	0.3817 (4)	0.6017 (4)	0.1612 (3)	0.0507 (14)	
H64A	0.3927	0.6129	0.2100	0.076*	
H64B	0.3607	0.5403	0.1400	0.076*	
H64C	0.3207	0.6211	0.1449	0.076*	
C65	0.4863 (4)	0.6460 (4)	0.0750 (2)	0.0481 (14)	
H65A	0.5620	0.6811	0.0721	0.072*	
H65B	0.4294	0.6692	0.0590	0.072*	
H65C	0.4680	0.5873	0.0471	0.072*	
N15	0.4854 (3)	0.6466 (3)	0.14468 (18)	0.0334 (9)	
O22	0.5179 (4)	0.2733 (4)	0.3899 (2)	0.0788 (15)	
O24	0.5010 (5)	0.2664 (4)	0.2505 (3)	0.0444 (19)	0.67831 (1100)
H24A	0.5084	0.2541	0.2869	0.067*	0.67831 (1100)
C69	0.6121 (8)	0.3057 (6)	0.2384 (4)	0.040 (2)	0.67831 (1100)
H69A	0.6086	0.3474	0.2141	0.060*	0.67831 (1100)
H69B	0.6361	0.2618	0.2115	0.060*	0.67831 (1100)
H69C	0.6671	0.3345	0.2813	0.060*	0.67831 (1100)
O24B	0.6205 (15)	0.3611 (15)	0.2170 (13)	0.132 (11)	0.32169 (1100)
H24B	0.6722	0.4030	0.2132	0.198*	0.32169 (1100)
C69B	0.669 (2)	0.3101 (19)	0.244 (2)	0.20 (3)	0.32169 (1100)
H69D	0.7283	0.3459	0.2846	0.300*	0.32169 (1100)
H69E	0.7035	0.2811	0.2114	0.300*	0.32169 (1100)
H69F	0.6103	0.2676	0.2567	0.300*	0.32169 (1100)
O25	0.415 (3)	0.255 (2)	0.2596 (11)	0.082 (17)	0.12419 (1500)
H25A	0.445 (3)	0.294 (5)	0.242 (2)	0.123*	0.12419 (1500)
H25B	0.463 (2)	0.262 (5)	0.2938 (16)	0.123*	0.12419 (1500)
O26	0.1530 (10)	0.8763 (16)	0.9555 (9)	0.051 (2)	0.56763 (700)
C70	0.1884 (10)	0.9115 (10)	0.9036 (8)	0.049 (3)	0.56763 (700)
H70A	0.1479	0.8701	0.8593	0.059*	0.56763 (700)
H70B	0.1694	0.9639	0.9066	0.059*	0.56763 (700)
C71	0.3076 (10)	0.9310 (14)	0.9103 (12)	0.069 (4)	0.56763 (700)
H71A	0.3475	0.9766	0.9520	0.103*	0.56763 (700)
H71B	0.3311	0.9499	0.8721	0.103*	0.56763 (700)
H71C	0.3267	0.8801	0.9115	0.103*	0.56763 (700)
C72	0.0321 (9)	0.8431 (7)	0.9482 (6)	0.052 (2)	0.56763 (700)
H72A	−0.0024	0.8068	0.9015	0.063*	0.56763 (700)
H72B	0.0144	0.8063	0.9782	0.063*	0.56763 (700)
C73	−0.0181 (9)	0.9049 (5)	0.9628 (5)	0.058 (2)	0.56763 (700)
H73A	0.0196	0.9442	1.0076	0.087*	0.56763 (700)
H73B	−0.0991	0.8775	0.9615	0.087*	0.56763 (700)
H73C	−0.0104	0.9367	0.9294	0.087*	0.56763 (700)
O26B	0.1695 (13)	0.885 (2)	0.9416 (13)	0.051 (2)	0.43237 (700)
C70B	0.2348 (15)	0.9210 (14)	0.8986 (10)	0.049 (3)	0.43237 (700)
H70C	0.2062	0.8825	0.8522	0.059*	0.43237 (700)
H70D	0.2262	0.9763	0.8986	0.059*	0.43237 (700)
C71B	0.3492 (14)	0.933 (2)	0.9193 (17)	0.069 (4)	0.43237 (700)
H71D	0.3718	0.9565	0.9683	0.103*	0.43237 (700)
H71E	0.3952	0.9732	0.8986	0.103*	0.43237 (700)
H71F	0.3613	0.8785	0.9057	0.103*	0.43237 (700)
C72B	0.0583 (11)	0.8893 (9)	0.9268 (7)	0.052 (2)	0.43237 (700)
H72C	0.0638	0.9497	0.9356	0.063*	0.43237 (700)
H72D	0.0236	0.8601	0.8787	0.063*	0.43237 (700)
C73B	−0.0124 (11)	0.8504 (11)	0.9665 (7)	0.058 (2)	0.43237 (700)
H73D	0.0229	0.8159	0.9866	0.087*	0.43237 (700)
H73E	−0.0872	0.8138	0.9381	0.087*	0.43237 (700)
H73F	−0.0218	0.8947	1.0020	0.087*	0.43237 (700)

### Atomic displacement parameters (Å^2^)

**Table:**

	*U*^11^	*U*^22^	*U*^33^	*U*^12^	*U*^13^	*U*^23^
C1	0.0182 (17)	0.0149 (17)	0.0152 (16)	0.0064 (14)	−0.0014 (13)	−0.0004 (13)
C2	0.0204 (18)	0.0166 (18)	0.0139 (16)	0.0087 (14)	−0.0030 (13)	−0.0020 (13)
C3	0.0247 (19)	0.024 (2)	0.0223 (18)	0.0154 (16)	0.0036 (15)	0.0030 (15)
C4	0.031 (2)	0.028 (2)	0.029 (2)	0.0200 (18)	0.0049 (17)	0.0014 (17)
C5	0.047 (3)	0.023 (2)	0.038 (2)	0.024 (2)	0.008 (2)	0.0046 (18)
C6	0.039 (2)	0.019 (2)	0.036 (2)	0.0143 (18)	0.0103 (19)	0.0080 (17)
C7	0.0227 (19)	0.0170 (18)	0.0185 (17)	0.0089 (15)	−0.0020 (14)	−0.0038 (14)
C8	0.0114 (16)	0.0150 (17)	0.0234 (18)	0.0050 (13)	0.0032 (13)	0.0061 (14)
C9	0.0156 (17)	0.0215 (19)	0.0167 (17)	0.0067 (14)	0.0011 (13)	0.0041 (14)
C10	0.0214 (19)	0.026 (2)	0.0265 (19)	0.0110 (16)	0.0044 (15)	0.0091 (16)
C11	0.028 (2)	0.039 (3)	0.029 (2)	0.0159 (19)	−0.0046 (17)	0.0097 (19)
C12	0.040 (3)	0.044 (3)	0.027 (2)	0.016 (2)	−0.0134 (19)	0.002 (2)
C13	0.043 (3)	0.039 (3)	0.025 (2)	0.021 (2)	−0.0104 (19)	−0.0050 (19)
C14	0.0230 (19)	0.026 (2)	0.0220 (19)	0.0106 (16)	−0.0042 (15)	0.0021 (16)
C15	0.0144 (16)	0.0179 (18)	0.0155 (16)	0.0071 (14)	0.0021 (13)	−0.0002 (13)
C16	0.0157 (17)	0.0179 (18)	0.0187 (17)	0.0051 (14)	0.0013 (14)	0.0012 (14)
C17	0.0223 (19)	0.027 (2)	0.0175 (17)	0.0078 (16)	0.0030 (15)	0.0022 (15)
C18	0.027 (2)	0.036 (2)	0.023 (2)	0.0057 (18)	0.0106 (17)	0.0071 (17)
C19	0.0163 (18)	0.040 (3)	0.028 (2)	0.0062 (17)	0.0068 (16)	0.0042 (18)
C20	0.0149 (17)	0.030 (2)	0.0221 (19)	0.0047 (16)	0.0011 (14)	0.0020 (16)
C21	0.0157 (17)	0.0207 (18)	0.0164 (16)	0.0052 (14)	0.0030 (13)	0.0022 (14)
C22	0.0184 (17)	0.0192 (18)	0.0151 (16)	0.0094 (15)	−0.0009 (13)	0.0006 (14)
C23	0.0231 (19)	0.0188 (19)	0.0224 (18)	0.0135 (15)	0.0024 (15)	0.0015 (15)
C24	0.029 (2)	0.028 (2)	0.030 (2)	0.0166 (18)	0.0070 (17)	0.0021 (17)
C25	0.039 (3)	0.028 (2)	0.055 (3)	0.024 (2)	0.016 (2)	0.006 (2)
C26	0.051 (3)	0.022 (2)	0.073 (4)	0.023 (2)	0.025 (3)	0.010 (2)
C27	0.045 (3)	0.022 (2)	0.059 (3)	0.018 (2)	0.022 (2)	0.015 (2)
C28	0.0240 (19)	0.0186 (19)	0.029 (2)	0.0135 (16)	0.0047 (16)	0.0041 (15)
C29	0.0152 (17)	0.0174 (17)	0.0190 (17)	0.0075 (14)	0.0041 (13)	0.0036 (14)
C30	0.0123 (16)	0.0206 (19)	0.0234 (18)	0.0046 (14)	0.0025 (14)	0.0054 (15)
C31	0.021 (2)	0.023 (2)	0.038 (2)	0.0069 (16)	0.0048 (17)	0.0066 (17)
C32	0.022 (2)	0.028 (2)	0.053 (3)	0.0008 (18)	0.008 (2)	0.012 (2)
C33	0.0144 (18)	0.037 (2)	0.039 (2)	0.0051 (17)	0.0059 (17)	0.0095 (19)
C34	0.0153 (18)	0.031 (2)	0.030 (2)	0.0090 (16)	0.0041 (15)	0.0079 (17)
C35	0.0208 (18)	0.0218 (19)	0.0184 (17)	0.0083 (15)	0.0040 (14)	0.0042 (14)
C36	0.0199 (18)	0.0170 (18)	0.0153 (16)	0.0050 (14)	−0.0016 (13)	0.0020 (14)
C37	0.025 (2)	0.023 (2)	0.061 (3)	0.0047 (18)	0.006 (2)	0.012 (2)
C38	0.0169 (17)	0.023 (2)	0.0206 (18)	0.0082 (15)	0.0053 (14)	0.0016 (15)
C39	0.027 (2)	0.029 (2)	0.041 (3)	0.0025 (19)	−0.0087 (19)	−0.002 (2)
C40	0.029 (2)	0.0207 (19)	0.0220 (18)	0.0083 (16)	0.0065 (16)	0.0047 (15)
C41	0.034 (2)	0.018 (2)	0.036 (2)	0.0083 (17)	0.0074 (19)	0.0034 (17)
C42	0.036 (2)	0.020 (2)	0.045 (3)	0.0047 (18)	0.011 (2)	0.0068 (19)
C43	0.045 (3)	0.035 (3)	0.044 (3)	0.010 (2)	0.026 (2)	0.011 (2)
C44	0.036 (3)	0.044 (3)	0.073 (4)	0.011 (2)	0.021 (3)	0.010 (3)
C45	0.0188 (18)	0.0205 (19)	0.0202 (18)	0.0089 (15)	−0.0005 (14)	−0.0003 (14)
C46	0.026 (2)	0.033 (2)	0.0175 (18)	0.0077 (17)	0.0063 (15)	0.0018 (16)
C47	0.035 (2)	0.042 (3)	0.0166 (19)	0.014 (2)	0.0026 (17)	−0.0013 (17)
C48	0.031 (2)	0.075 (4)	0.033 (2)	0.024 (3)	−0.013 (2)	−0.018 (2)
C49	0.081 (5)	0.116 (6)	0.075 (5)	−0.028 (4)	0.001 (4)	0.024 (5)
C50	0.0230 (19)	0.026 (2)	0.0247 (19)	0.0081 (16)	0.0063 (16)	0.0083 (16)
C51	0.037 (2)	0.033 (2)	0.027 (2)	0.017 (2)	0.0089 (18)	0.0061 (18)
C52	0.048 (3)	0.034 (2)	0.024 (2)	0.016 (2)	0.0058 (19)	0.0020 (18)
C53	0.031 (2)	0.054 (3)	0.031 (2)	0.014 (2)	−0.0006 (19)	0.018 (2)
C54	0.043 (3)	0.071 (4)	0.044 (3)	0.028 (3)	0.010 (2)	0.026 (3)
C55	0.026 (2)	0.021 (2)	0.0176 (17)	0.0087 (16)	−0.0018 (15)	−0.0051 (15)
C56	0.023 (2)	0.027 (2)	0.0199 (18)	0.0122 (17)	−0.0036 (15)	−0.0068 (16)
C57	0.032 (2)	0.026 (2)	0.0183 (18)	0.0135 (18)	−0.0049 (16)	−0.0082 (16)
C58	0.074 (4)	0.027 (2)	0.033 (2)	0.028 (3)	−0.008 (2)	−0.002 (2)
C59	0.112 (6)	0.031 (3)	0.044 (3)	0.022 (3)	0.023 (3)	0.013 (2)
C60	0.027 (2)	0.025 (2)	0.0193 (18)	0.0109 (17)	0.0016 (15)	−0.0045 (15)
C61	0.048 (3)	0.074 (4)	0.024 (2)	0.014 (3)	0.001 (2)	−0.002 (2)
C62	0.027 (2)	0.052 (3)	0.042 (3)	0.009 (2)	−0.007 (2)	−0.014 (2)
C66	0.050 (4)	0.092 (5)	0.051 (4)	0.020 (4)	0.001 (3)	0.011 (3)
C67	0.059 (4)	0.061 (4)	0.094 (6)	0.013 (3)	0.006 (4)	−0.004 (4)
C68	0.076 (4)	0.086 (5)	0.047 (3)	0.043 (4)	0.018 (3)	0.017 (3)
Mn1	0.0120 (2)	0.0129 (3)	0.0137 (2)	0.0055 (2)	0.00118 (19)	0.00076 (19)
Mn2	0.0186 (3)	0.0123 (3)	0.0167 (3)	0.0070 (2)	0.0027 (2)	0.0014 (2)
Mn3	0.0145 (3)	0.0161 (3)	0.0194 (3)	0.0084 (2)	0.0055 (2)	0.0035 (2)
Mn4	0.0160 (3)	0.0133 (3)	0.0207 (3)	0.0072 (2)	0.0051 (2)	0.0043 (2)
Mn5	0.0103 (2)	0.0181 (3)	0.0145 (2)	0.0044 (2)	−0.00008 (19)	0.0016 (2)
Mn6	0.0154 (3)	0.0165 (3)	0.0144 (3)	0.0073 (2)	−0.0043 (2)	−0.0044 (2)
N1	0.0192 (15)	0.0108 (14)	0.0165 (14)	0.0069 (12)	0.0010 (11)	0.0022 (11)
N2	0.0164 (14)	0.0156 (15)	0.0111 (13)	0.0055 (12)	−0.0007 (11)	−0.0007 (11)
N3	0.0085 (13)	0.0148 (15)	0.0190 (14)	0.0021 (11)	−0.0012 (11)	−0.0007 (12)
N4	0.0141 (14)	0.0126 (14)	0.0181 (14)	0.0044 (11)	0.0024 (11)	0.0026 (11)
N5	0.0103 (13)	0.0183 (15)	0.0180 (14)	0.0062 (11)	0.0035 (11)	0.0031 (12)
N6	0.0212 (16)	0.0165 (16)	0.0219 (15)	0.0058 (13)	0.0023 (13)	0.0033 (12)
N7	0.0310 (19)	0.0230 (18)	0.0323 (19)	0.0048 (15)	0.0117 (15)	0.0072 (15)
N8	0.0165 (15)	0.0201 (16)	0.0178 (14)	0.0080 (12)	0.0013 (12)	0.0026 (12)
N9	0.0269 (18)	0.0290 (19)	0.0208 (16)	0.0152 (15)	−0.0064 (14)	−0.0067 (14)
N10	0.0206 (16)	0.0218 (17)	0.0214 (16)	0.0067 (13)	0.0046 (13)	0.0059 (13)
N11	0.0237 (17)	0.036 (2)	0.0219 (16)	0.0079 (15)	0.0031 (14)	0.0119 (15)
N12	0.0176 (15)	0.0177 (16)	0.0157 (14)	0.0066 (12)	−0.0029 (12)	−0.0051 (12)
N13	0.037 (2)	0.0232 (18)	0.0223 (16)	0.0166 (15)	−0.0021 (14)	−0.0030 (14)
N14	0.0277 (18)	0.032 (2)	0.0218 (17)	0.0106 (15)	−0.0009 (14)	−0.0048 (14)
N16	0.051 (3)	0.056 (3)	0.052 (3)	0.016 (2)	0.010 (2)	0.009 (2)
O1	0.0162 (12)	0.0136 (12)	0.0199 (12)	0.0084 (10)	0.0040 (10)	0.0046 (10)
O2	0.0184 (12)	0.0194 (13)	0.0232 (13)	0.0106 (11)	0.0065 (10)	0.0047 (11)
O3	0.0235 (14)	0.0144 (13)	0.0294 (14)	0.0086 (11)	0.0064 (11)	0.0039 (11)
O4	0.0211 (13)	0.0142 (12)	0.0123 (11)	0.0073 (10)	0.0009 (10)	−0.0013 (9)
O5	0.0188 (13)	0.0204 (13)	0.0200 (12)	0.0098 (11)	0.0024 (10)	0.0042 (10)
O6	0.0363 (16)	0.0292 (16)	0.0212 (14)	0.0203 (13)	−0.0119 (12)	−0.0075 (12)
O7	0.0105 (11)	0.0173 (13)	0.0158 (12)	0.0049 (10)	−0.0006 (9)	−0.0009 (10)
O8	0.0169 (12)	0.0233 (14)	0.0128 (11)	0.0063 (10)	−0.0005 (9)	0.0000 (10)
O9	0.0120 (12)	0.0314 (15)	0.0177 (12)	0.0032 (11)	−0.0003 (10)	0.0052 (11)
O10	0.0147 (12)	0.0127 (12)	0.0180 (12)	0.0052 (10)	0.0024 (9)	0.0012 (9)
O11	0.0159 (12)	0.0208 (14)	0.0256 (13)	0.0091 (11)	0.0059 (10)	0.0035 (11)
O12	0.0297 (15)	0.0174 (13)	0.0303 (15)	0.0136 (11)	0.0117 (12)	0.0077 (11)
O13	0.0121 (11)	0.0165 (12)	0.0175 (12)	0.0067 (10)	0.0032 (9)	0.0030 (10)
O14	0.0185 (13)	0.0169 (13)	0.0262 (13)	0.0071 (10)	0.0070 (11)	0.0067 (11)
O15	0.0194 (13)	0.0233 (14)	0.0262 (14)	0.0101 (11)	0.0110 (11)	0.0069 (11)
O16	0.0232 (13)	0.0187 (13)	0.0192 (13)	0.0097 (11)	−0.0002 (10)	0.0004 (10)
O17	0.0158 (12)	0.0167 (13)	0.0195 (12)	0.0061 (10)	0.0010 (10)	0.0020 (10)
O18	0.0154 (12)	0.0210 (14)	0.0190 (12)	0.0063 (10)	0.0009 (10)	0.0003 (10)
O19	0.0287 (15)	0.0189 (14)	0.0239 (14)	0.0083 (12)	0.0035 (11)	0.0025 (11)
O20	0.0238 (14)	0.0272 (15)	0.0206 (13)	0.0086 (12)	0.0016 (11)	0.0004 (11)
O21	0.0215 (14)	0.0301 (16)	0.0295 (15)	0.0100 (12)	0.0004 (12)	0.0039 (12)
C63	0.0184 (19)	0.027 (2)	0.031 (2)	0.0115 (16)	−0.0032 (16)	0.0000 (17)
C64	0.036 (3)	0.081 (4)	0.033 (3)	0.020 (3)	0.008 (2)	0.013 (3)
C65	0.029 (2)	0.069 (4)	0.030 (2)	0.010 (2)	0.002 (2)	−0.003 (2)
N15	0.0189 (17)	0.045 (2)	0.0253 (18)	0.0117 (16)	−0.0036 (14)	−0.0079 (16)
O22	0.063 (3)	0.118 (4)	0.058 (3)	0.044 (3)	0.015 (2)	0.014 (3)
O24	0.049 (4)	0.044 (3)	0.034 (3)	0.015 (3)	0.000 (2)	0.007 (2)
C69	0.041 (5)	0.043 (5)	0.047 (4)	0.024 (4)	0.012 (4)	0.022 (4)
O24B	0.059 (11)	0.108 (18)	0.16 (2)	−0.008 (11)	0.026 (13)	−0.034 (17)
C69B	0.060 (19)	0.065 (19)	0.51 (10)	0.025 (16)	0.08 (3)	0.15 (4)
O25	0.06 (3)	0.06 (2)	0.08 (3)	−0.009 (17)	0.03 (2)	−0.020 (18)
O26	0.048 (4)	0.066 (6)	0.040 (8)	0.020 (5)	0.023 (3)	0.010 (5)
C70	0.034 (8)	0.054 (5)	0.061 (5)	0.017 (7)	0.013 (7)	0.016 (4)
C71	0.025 (10)	0.054 (5)	0.131 (10)	0.009 (9)	0.024 (11)	0.034 (6)
C72	0.066 (6)	0.058 (5)	0.063 (5)	0.038 (5)	0.034 (4)	0.040 (4)
C73	0.064 (5)	0.078 (7)	0.047 (5)	0.039 (6)	0.010 (4)	0.027 (5)
O26B	0.048 (4)	0.066 (6)	0.040 (8)	0.020 (5)	0.023 (3)	0.010 (5)
C70B	0.034 (8)	0.054 (5)	0.061 (5)	0.017 (7)	0.013 (7)	0.016 (4)
C71B	0.025 (10)	0.054 (5)	0.131 (10)	0.009 (9)	0.024 (11)	0.034 (6)
C72B	0.066 (6)	0.058 (5)	0.063 (5)	0.038 (5)	0.034 (4)	0.040 (4)
C73B	0.064 (5)	0.078 (7)	0.047 (5)	0.039 (6)	0.010 (4)	0.027 (5)

### Geometric parameters (Å, °)

**Table:**

C1—O2	1.298 (4)	C56—C57	1.347 (6)
C1—N1	1.316 (4)	C56—N12	1.383 (4)
C1—C2	1.462 (5)	C56—H56	0.9500
C2—C3	1.403 (5)	C57—N13	1.368 (5)
C2—C7	1.410 (6)	C57—H57	0.9500
C3—C4	1.382 (5)	C58—N13	1.483 (6)
C3—H3	0.9500	C58—C59	1.504 (8)
C4—C5	1.386 (7)	C58—H58A	0.9900
C4—H4	0.9500	C58—H58B	0.9900
C5—C6	1.380 (6)	C59—H59A	0.9800
C5—H5	0.9500	C59—H59B	0.9800
C6—C7	1.408 (5)	C59—H59C	0.9800
C6—H6	0.9500	C60—O20	1.240 (5)
C7—O3	1.332 (4)	C60—N14	1.327 (5)
C8—O5	1.279 (4)	C60—H60	0.9500
C8—N2	1.322 (4)	C61—N14	1.434 (6)
C8—C9	1.469 (5)	C61—H61A	0.9800
C9—C10	1.404 (5)	C61—H61B	0.9800
C9—C14	1.406 (5)	C61—H61C	0.9800
C10—C11	1.376 (6)	C62—N14	1.462 (6)
C10—H10	0.9500	C62—H62A	0.9800
C11—C12	1.397 (6)	C62—H62B	0.9800
C11—H11	0.9500	C62—H62C	0.9800
C12—C13	1.374 (6)	C66—O22	1.247 (8)
C12—H12	0.9500	C66—N16	1.326 (8)
C13—C14	1.402 (5)	C66—H66	0.9500
C13—H13	0.9500	C67—N16	1.449 (8)
C14—O6	1.333 (5)	C67—H67A	0.9800
C15—O8	1.288 (4)	C67—H67B	0.9800
C15—N3	1.309 (5)	C67—H67C	0.9800
C15—C16	1.476 (5)	C68—N16	1.471 (7)
C16—C17	1.406 (5)	C68—H68A	0.9800
C16—C21	1.407 (5)	C68—H68B	0.9800
C17—C18	1.377 (6)	C68—H68C	0.9800
C17—H17	0.9500	Mn1—O18	2.205 (3)
C18—C19	1.390 (6)	Mn1—O7	2.229 (2)
C18—H18	0.9500	Mn1—O13	2.235 (2)
C19—C20	1.378 (6)	Mn1—O1	2.243 (2)
C19—H19	0.9500	Mn1—O17	2.263 (3)
C20—C21	1.403 (5)	Mn1—O4	2.264 (3)
C20—H20	0.9500	Mn1—O10	2.265 (2)
C21—O9	1.338 (4)	Mn2—O3	1.860 (3)
C22—O11	1.275 (5)	Mn2—O4	1.901 (2)
C22—N4	1.322 (4)	Mn2—N1	2.001 (3)
C22—C23	1.476 (5)	Mn2—N6	2.059 (3)
C23—C28	1.405 (6)	Mn2—O5	2.141 (3)
C23—C24	1.408 (5)	Mn2—O16	2.243 (3)
C24—C25	1.377 (6)	Mn3—O15	1.862 (3)
C24—H24	0.9500	Mn3—O1	1.917 (3)
C25—C26	1.382 (7)	Mn3—O2	1.951 (3)
C25—H25	0.9500	Mn3—N5	1.955 (3)
C26—C27	1.385 (6)	Mn3—O21	2.192 (3)
C26—H26	0.9500	Mn3—O20	2.273 (3)
C27—C28	1.399 (6)	Mn4—O12	1.872 (3)
C27—H27	0.9500	Mn4—O13	1.936 (2)
C28—O12	1.329 (4)	Mn4—O14	1.938 (3)
C29—O14	1.293 (4)	Mn4—N4	1.941 (3)
C29—N5	1.314 (5)	Mn4—O19	2.235 (3)
C29—C30	1.470 (5)	Mn4—N10	2.276 (3)
C30—C31	1.397 (5)	Mn5—O9	1.849 (3)
C30—C35	1.417 (5)	Mn5—O10	1.912 (2)
C31—C32	1.375 (6)	Mn5—N3	1.975 (3)
C31—H31	0.9500	Mn5—N8	2.042 (3)
C32—C33	1.395 (6)	Mn5—O11	2.148 (3)
C32—H32	0.9500	Mn5—O17	2.398 (2)
C33—C34	1.372 (6)	Mn6—O6	1.841 (3)
C33—H33	0.9500	Mn6—O7	1.890 (2)
C34—C35	1.394 (5)	Mn6—N2	1.969 (3)
C34—H34	0.9500	Mn6—N12	2.032 (3)
C35—O15	1.332 (5)	Mn6—O8	2.112 (3)
C36—O16	1.253 (4)	N1—O1	1.403 (4)
C36—O17	1.267 (4)	N2—O4	1.411 (3)
C36—C37	1.507 (6)	N3—O7	1.403 (4)
C37—H37A	0.9800	N4—O10	1.392 (4)
C37—H37B	0.9800	N5—O13	1.409 (3)
C37—H37C	0.9800	O21—C63	1.242 (5)
C38—O19	1.250 (5)	C63—N15	1.328 (5)
C38—O18	1.266 (5)	C63—H63	0.9500
C38—C39	1.515 (5)	C64—N15	1.433 (6)
C39—H39A	0.9800	C64—H64A	0.9800
C39—H39B	0.9800	C64—H64B	0.9800
C39—H39C	0.9800	C64—H64C	0.9800
C40—N6	1.324 (5)	C65—N15	1.463 (6)
C40—N7	1.346 (5)	C65—H65A	0.9800
C40—H40	0.9500	C65—H65B	0.9800
C41—C42	1.342 (6)	C65—H65C	0.9800
C41—N6	1.380 (5)	O24—C69	1.442 (11)
C41—H41	0.9500	O24—H24A	0.8400
C42—N7	1.367 (5)	O24—H25A	0.98 (7)
C42—H42	0.9500	O24—H25B	1.09 (3)
C43—N7	1.471 (6)	C69—H69A	0.9800
C43—C44	1.505 (7)	C69—H69B	0.9800
C43—H43A	0.9900	C69—H69C	0.9800
C43—H43B	0.9900	O24B—C69B	1.40 (2)
C44—H44A	0.9800	O24B—H24B	0.8400
C44—H44B	0.9800	C69B—H69D	0.9800
C44—H44C	0.9800	C69B—H69E	0.9800
C45—N8	1.317 (5)	C69B—H69F	0.9800
C45—N9	1.349 (5)	O25—H25A	0.84 (2)
C45—H45	0.9500	O25—H25A	0.84 (2)
C46—C47	1.349 (6)	O25—H25B	0.835 (18)
C46—N8	1.377 (5)	O26—C70	1.423 (11)
C46—H46	0.9500	O26—C72	1.429 (13)
C47—N9	1.366 (6)	C70—C71	1.417 (11)
C47—H47	0.9500	C70—H70A	0.9900
C48—C49	1.453 (10)	C70—H70B	0.9900
C48—N9	1.473 (5)	C71—H71A	0.9800
C48—H48A	0.9900	C71—H71B	0.9800
C48—H48B	0.9900	C71—H71C	0.9800
C49—H49A	0.9800	C72—C73	1.388 (10)
C49—H49B	0.9800	C72—H72A	0.9900
C49—H49C	0.9800	C72—H72B	0.9900
C50—N10	1.313 (5)	C73—H73A	0.9800
C50—N11	1.347 (5)	C73—H73B	0.9800
C50—H50	0.9500	C73—H73C	0.9800
C51—C52	1.355 (6)	O26B—C70B	1.421 (14)
C51—N10	1.377 (5)	O26B—C72B	1.424 (13)
C51—H51	0.9500	C70B—C71B	1.387 (13)
C52—N11	1.381 (6)	C70B—H70C	0.9900
C52—H52	0.9500	C70B—H70D	0.9900
C53—N11	1.467 (5)	C71B—H71D	0.9800
C53—C54	1.467 (7)	C71B—H71E	0.9800
C53—H53A	0.9900	C71B—H71F	0.9800
C53—H53B	0.9900	C72B—C73B	1.422 (11)
C54—H54A	0.9800	C72B—H72C	0.9900
C54—H54B	0.9800	C72B—H72D	0.9900
C54—H54C	0.9800	C73B—H73D	0.9800
C55—N12	1.318 (5)	C73B—H73E	0.9800
C55—N13	1.335 (5)	C73B—H73F	0.9800
C55—H55	0.9500		
			
O2—C1—N1	120.4 (3)	O18—Mn1—O13	87.02 (10)
O2—C1—C2	119.4 (3)	O7—Mn1—O13	127.19 (9)
N1—C1—C2	120.2 (3)	O18—Mn1—O1	93.10 (9)
C3—C2—C7	119.0 (3)	O7—Mn1—O1	147.92 (9)
C3—C2—C1	118.3 (3)	O13—Mn1—O1	77.71 (9)
C7—C2—C1	122.7 (3)	O18—Mn1—O17	157.00 (9)
C4—C3—C2	121.8 (4)	O7—Mn1—O17	86.99 (9)
C4—C3—H3	119.1	O13—Mn1—O17	112.73 (9)
C2—C3—H3	119.1	O1—Mn1—O17	102.30 (9)
C3—C4—C5	119.0 (4)	O18—Mn1—O4	88.74 (9)
C3—C4—H4	120.5	O7—Mn1—O4	75.21 (9)
C5—C4—H4	120.5	O13—Mn1—O4	153.77 (9)
C6—C5—C4	120.7 (4)	O1—Mn1—O4	76.70 (9)
C6—C5—H5	119.7	O17—Mn1—O4	78.48 (9)
C4—C5—H5	119.7	O18—Mn1—O10	113.49 (9)
C5—C6—C7	121.1 (4)	O7—Mn1—O10	73.78 (9)
C5—C6—H6	119.4	O13—Mn1—O10	72.20 (9)
C7—C6—H6	119.4	O1—Mn1—O10	138.01 (9)
O3—C7—C6	117.4 (4)	O17—Mn1—O10	64.99 (9)
O3—C7—C2	124.1 (3)	O4—Mn1—O10	132.60 (9)
C6—C7—C2	118.4 (4)	O3—Mn2—O4	177.37 (11)
O5—C8—N2	120.8 (3)	O3—Mn2—N1	88.86 (12)
O5—C8—C9	120.1 (3)	O4—Mn2—N1	90.97 (11)
N2—C8—C9	119.1 (3)	O3—Mn2—N6	89.00 (12)
C10—C9—C14	118.3 (3)	O4—Mn2—N6	91.59 (12)
C10—C9—C8	118.3 (3)	N1—Mn2—N6	170.25 (12)
C14—C9—C8	123.4 (3)	O3—Mn2—O5	99.06 (11)
C11—C10—C9	121.7 (4)	O4—Mn2—O5	78.37 (10)
C11—C10—H10	119.1	N1—Mn2—O5	98.38 (11)
C9—C10—H10	119.1	N6—Mn2—O5	91.35 (11)
C10—C11—C12	119.6 (4)	O3—Mn2—O16	97.26 (11)
C10—C11—H11	120.2	O4—Mn2—O16	85.36 (10)
C12—C11—H11	120.2	N1—Mn2—O16	88.14 (11)
C13—C12—C11	119.8 (4)	N6—Mn2—O16	82.69 (11)
C13—C12—H12	120.1	O5—Mn2—O16	162.52 (9)
C11—C12—H12	120.1	O15—Mn3—O1	179.39 (11)
C12—C13—C14	121.2 (4)	O15—Mn3—O2	97.41 (11)
C12—C13—H13	119.4	O1—Mn3—O2	81.99 (10)
C14—C13—H13	119.4	O15—Mn3—N5	90.10 (12)
O6—C14—C13	117.1 (4)	O1—Mn3—N5	90.51 (11)
O6—C14—C9	123.5 (3)	O2—Mn3—N5	172.17 (12)
C13—C14—C9	119.4 (4)	O15—Mn3—O21	88.05 (12)
O8—C15—N3	121.1 (3)	O1—Mn3—O21	91.88 (11)
O8—C15—C16	120.2 (3)	O2—Mn3—O21	89.67 (11)
N3—C15—C16	118.6 (3)	N5—Mn3—O21	92.83 (11)
C17—C16—C21	119.1 (3)	O15—Mn3—O20	90.96 (11)
C17—C16—C15	118.6 (3)	O1—Mn3—O20	89.09 (10)
C21—C16—C15	122.3 (3)	O2—Mn3—O20	88.31 (11)
C18—C17—C16	121.1 (4)	N5—Mn3—O20	89.34 (11)
C18—C17—H17	119.4	O21—Mn3—O20	177.61 (10)
C16—C17—H17	119.4	O12—Mn4—O13	177.47 (11)
C17—C18—C19	119.7 (4)	O12—Mn4—O14	95.31 (11)
C17—C18—H18	120.1	O13—Mn4—O14	82.24 (10)
C19—C18—H18	120.1	O12—Mn4—N4	89.32 (12)
C20—C19—C18	120.1 (4)	O13—Mn4—N4	93.16 (11)
C20—C19—H19	120.0	O14—Mn4—N4	174.60 (12)
C18—C19—H19	120.0	O12—Mn4—O19	92.92 (11)
C19—C20—C21	121.3 (4)	O13—Mn4—O19	87.52 (10)
C19—C20—H20	119.3	O14—Mn4—O19	84.70 (11)
C21—C20—H20	119.3	N4—Mn4—O19	92.31 (11)
O9—C21—C20	116.8 (3)	O12—Mn4—N10	90.88 (12)
O9—C21—C16	124.6 (3)	O13—Mn4—N10	88.56 (11)
C20—C21—C16	118.6 (3)	O14—Mn4—N10	92.21 (11)
O11—C22—N4	120.9 (3)	N4—Mn4—N10	90.50 (12)
O11—C22—C23	121.3 (3)	O19—Mn4—N10	175.30 (11)
N4—C22—C23	117.8 (3)	O9—Mn5—O10	176.63 (11)
C28—C23—C24	119.4 (4)	O9—Mn5—N3	88.23 (12)
C28—C23—C22	123.1 (3)	O10—Mn5—N3	93.31 (11)
C24—C23—C22	117.5 (4)	O9—Mn5—N8	87.30 (12)
C25—C24—C23	121.3 (4)	O10—Mn5—N8	90.69 (11)
C25—C24—H24	119.3	N3—Mn5—N8	169.82 (13)
C23—C24—H24	119.3	O9—Mn5—O11	104.93 (11)
C24—C25—C26	119.2 (4)	O10—Mn5—O11	77.94 (10)
C24—C25—H25	120.4	N3—Mn5—O11	94.58 (11)
C26—C25—H25	120.4	N8—Mn5—O11	95.41 (11)
C25—C26—C27	120.7 (4)	O9—Mn5—O17	109.67 (11)
C25—C26—H26	119.6	O10—Mn5—O17	67.64 (9)
C27—C26—H26	119.6	N3—Mn5—O17	81.70 (10)
C26—C27—C28	121.0 (4)	N8—Mn5—O17	91.20 (10)
C26—C27—H27	119.5	O11—Mn5—O17	145.02 (9)
C28—C27—H27	119.5	O6—Mn6—O7	176.15 (12)
O12—C28—C27	116.3 (4)	O6—Mn6—N2	89.00 (12)
O12—C28—C23	125.2 (3)	O7—Mn6—N2	94.26 (11)
C27—C28—C23	118.4 (4)	O6—Mn6—N12	87.48 (12)
O14—C29—N5	121.2 (3)	O7—Mn6—N12	88.78 (11)
O14—C29—C30	119.4 (3)	N2—Mn6—N12	161.73 (13)
N5—C29—C30	119.4 (3)	O6—Mn6—O8	103.09 (13)
C31—C30—C35	119.5 (3)	O7—Mn6—O8	78.56 (10)
C31—C30—C29	118.0 (3)	N2—Mn6—O8	97.25 (11)
C35—C30—C29	122.5 (3)	N12—Mn6—O8	101.01 (11)
C32—C31—C30	121.2 (4)	C1—N1—O1	113.5 (3)
C32—C31—H31	119.4	C1—N1—Mn2	129.7 (2)
C30—C31—H31	119.4	O1—N1—Mn2	116.9 (2)
C31—C32—C33	119.4 (4)	C8—N2—O4	114.1 (3)
C31—C32—H32	120.3	C8—N2—Mn6	132.1 (2)
C33—C32—H32	120.3	O4—N2—Mn6	113.7 (2)
C34—C33—C32	120.1 (4)	C15—N3—O7	113.4 (3)
C34—C33—H33	119.9	C15—N3—Mn5	133.4 (2)
C32—C33—H33	119.9	O7—N3—Mn5	112.7 (2)
C33—C34—C35	121.8 (4)	C22—N4—O10	114.5 (3)
C33—C34—H34	119.1	C22—N4—Mn4	133.7 (3)
C35—C34—H34	119.1	O10—N4—Mn4	111.80 (19)
O15—C35—C34	119.5 (3)	C29—N5—O13	113.3 (3)
O15—C35—C30	122.5 (3)	C29—N5—Mn3	128.1 (2)
C34—C35—C30	118.0 (4)	O13—N5—Mn3	118.2 (2)
O16—C36—O17	123.0 (3)	C40—N6—C41	106.3 (3)
O16—C36—C37	119.5 (3)	C40—N6—Mn2	127.7 (3)
O17—C36—C37	117.6 (3)	C41—N6—Mn2	124.8 (3)
C36—C37—H37A	109.5	C40—N7—C42	107.4 (4)
C36—C37—H37B	109.5	C40—N7—C43	126.8 (4)
H37A—C37—H37B	109.5	C42—N7—C43	125.8 (4)
C36—C37—H37C	109.5	C45—N8—C46	106.7 (3)
H37A—C37—H37C	109.5	C45—N8—Mn5	126.9 (3)
H37B—C37—H37C	109.5	C46—N8—Mn5	126.4 (3)
O19—C38—O18	125.7 (3)	C45—N9—C47	107.5 (3)
O19—C38—C39	117.4 (4)	C45—N9—C48	126.3 (4)
O18—C38—C39	116.8 (3)	C47—N9—C48	126.2 (4)
C38—C39—H39A	109.5	C50—N10—C51	105.2 (3)
C38—C39—H39B	109.5	C50—N10—Mn4	124.9 (3)
H39A—C39—H39B	109.5	C51—N10—Mn4	129.4 (3)
C38—C39—H39C	109.5	C50—N11—C52	106.1 (3)
H39A—C39—H39C	109.5	C50—N11—C53	128.6 (4)
H39B—C39—H39C	109.5	C52—N11—C53	125.3 (4)
N6—C40—N7	110.4 (3)	C55—N12—C56	106.2 (3)
N6—C40—H40	124.8	C55—N12—Mn6	126.6 (2)
N7—C40—H40	124.8	C56—N12—Mn6	127.0 (3)
C42—C41—N6	108.9 (4)	C55—N13—C57	107.3 (3)
C42—C41—H41	125.5	C55—N13—C58	126.4 (4)
N6—C41—H41	125.5	C57—N13—C58	126.0 (3)
C41—C42—N7	107.1 (4)	C60—N14—C61	121.0 (4)
C41—C42—H42	126.5	C60—N14—C62	121.0 (4)
N7—C42—H42	126.5	C61—N14—C62	118.1 (4)
N7—C43—C44	111.6 (4)	C66—N16—C67	120.0 (6)
N7—C43—H43A	109.3	C66—N16—C68	121.9 (6)
C44—C43—H43A	109.3	C67—N16—C68	118.0 (5)
N7—C43—H43B	109.3	N1—O1—Mn3	112.10 (19)
C44—C43—H43B	109.3	N1—O1—Mn1	125.31 (19)
H43A—C43—H43B	108.0	Mn3—O1—Mn1	122.37 (12)
C43—C44—H44A	109.5	C1—O2—Mn3	111.7 (2)
C43—C44—H44B	109.5	C7—O3—Mn2	128.4 (2)
H44A—C44—H44B	109.5	N2—O4—Mn2	115.97 (19)
C43—C44—H44C	109.5	N2—O4—Mn1	113.29 (18)
H44A—C44—H44C	109.5	Mn2—O4—Mn1	119.61 (11)
H44B—C44—H44C	109.5	C8—O5—Mn2	109.5 (2)
N8—C45—N9	110.2 (3)	C14—O6—Mn6	132.6 (2)
N8—C45—H45	124.9	N3—O7—Mn6	117.0 (2)
N9—C45—H45	124.9	N3—O7—Mn1	114.51 (18)
C47—C46—N8	108.8 (4)	Mn6—O7—Mn1	111.00 (11)
C47—C46—H46	125.6	C15—O8—Mn6	109.9 (2)
N8—C46—H46	125.6	C21—O9—Mn5	131.9 (2)
C46—C47—N9	106.8 (4)	N4—O10—Mn5	116.74 (19)
C46—C47—H47	126.6	N4—O10—Mn1	115.60 (18)
N9—C47—H47	126.6	Mn5—O10—Mn1	108.37 (11)
C49—C48—N9	111.8 (5)	C22—O11—Mn5	109.9 (2)
C49—C48—H48A	109.3	C28—O12—Mn4	129.7 (3)
N9—C48—H48A	109.3	N5—O13—Mn4	110.59 (19)
C49—C48—H48B	109.3	N5—O13—Mn1	116.94 (18)
N9—C48—H48B	109.3	Mn4—O13—Mn1	112.59 (11)
H48A—C48—H48B	107.9	C29—O14—Mn4	111.3 (2)
C48—C49—H49A	109.5	C35—O15—Mn3	123.3 (2)
C48—C49—H49B	109.5	C36—O16—Mn2	123.5 (2)
H49A—C49—H49B	109.5	C36—O17—Mn1	125.9 (2)
C48—C49—H49C	109.5	C36—O17—Mn5	132.5 (2)
H49A—C49—H49C	109.5	Mn1—O17—Mn5	93.39 (9)
H49B—C49—H49C	109.5	C38—O18—Mn1	127.5 (2)
N10—C50—N11	112.5 (4)	C38—O19—Mn4	136.3 (2)
N10—C50—H50	123.8	C60—O20—Mn3	122.1 (2)
N11—C50—H50	123.8	C63—O21—Mn3	132.1 (3)
C52—C51—N10	109.7 (4)	O21—C63—N15	120.5 (4)
C52—C51—H51	125.1	O21—C63—H63	119.8
N10—C51—H51	125.1	N15—C63—H63	119.8
C51—C52—N11	106.5 (4)	N15—C64—H64A	109.5
C51—C52—H52	126.8	N15—C64—H64B	109.5
N11—C52—H52	126.8	H64A—C64—H64B	109.5
N11—C53—C54	112.3 (4)	N15—C64—H64C	109.5
N11—C53—H53A	109.2	H64A—C64—H64C	109.5
C54—C53—H53A	109.2	H64B—C64—H64C	109.5
N11—C53—H53B	109.2	N15—C65—H65A	109.5
C54—C53—H53B	109.2	N15—C65—H65B	109.5
H53A—C53—H53B	107.9	H65A—C65—H65B	109.5
C53—C54—H54A	109.5	N15—C65—H65C	109.5
C53—C54—H54B	109.5	H65A—C65—H65C	109.5
H54A—C54—H54B	109.5	H65B—C65—H65C	109.5
C53—C54—H54C	109.5	C63—N15—C64	119.3 (4)
H54A—C54—H54C	109.5	C63—N15—C65	121.4 (4)
H54B—C54—H54C	109.5	C64—N15—C65	119.2 (4)
N12—C55—N13	111.0 (3)	C69—O24—H25A	115.7 (18)
N12—C55—H55	124.5	H24A—O24—H25A	117.5
N13—C55—H55	124.5	C69—O24—H25B	137.2 (19)
C57—C56—N12	108.5 (4)	H25A—O24—H25B	81 (4)
C57—C56—H56	125.8	C69B—O24B—H24B	109.5
N12—C56—H56	125.8	O24B—C69B—H69D	109.5
C56—C57—N13	107.0 (3)	O24B—C69B—H69E	109.5
C56—C57—H57	126.5	H69D—C69B—H69E	109.5
N13—C57—H57	126.5	O24B—C69B—H69F	109.5
N13—C58—C59	111.5 (4)	H69D—C69B—H69F	109.5
N13—C58—H58A	109.3	H69E—C69B—H69F	109.5
C59—C58—H58A	109.3	H25A—O25—H25B	107 (3)
N13—C58—H58B	109.3	H25A—O25—H25B	107 (3)
C59—C58—H58B	109.3	C70—O26—C72	114.0 (10)
H58A—C58—H58B	108.0	C71—C70—O26	109.2 (10)
C58—C59—H59A	109.5	C71—C70—H70A	109.8
C58—C59—H59B	109.5	O26—C70—H70A	109.8
H59A—C59—H59B	109.5	C71—C70—H70B	109.8
C58—C59—H59C	109.5	O26—C70—H70B	109.8
H59A—C59—H59C	109.5	H70A—C70—H70B	108.3
H59B—C59—H59C	109.5	C73—C72—O26	114.4 (12)
O20—C60—N14	123.9 (4)	C73—C72—H72A	108.7
O20—C60—H60	118.0	O26—C72—H72A	108.7
N14—C60—H60	118.0	C73—C72—H72B	108.7
N14—C61—H61A	109.5	O26—C72—H72B	108.7
N14—C61—H61B	109.5	H72A—C72—H72B	107.6
H61A—C61—H61B	109.5	C70B—O26B—C72B	108.9 (14)
N14—C61—H61C	109.5	C71B—C70B—O26B	110.9 (13)
H61A—C61—H61C	109.5	C71B—C70B—H70C	109.5
H61B—C61—H61C	109.5	O26B—C70B—H70C	109.5
N14—C62—H62A	109.5	C71B—C70B—H70D	109.5
N14—C62—H62B	109.5	O26B—C70B—H70D	109.5
H62A—C62—H62B	109.5	H70C—C70B—H70D	108.0
N14—C62—H62C	109.5	C70B—C71B—H71D	109.5
H62A—C62—H62C	109.5	C70B—C71B—H71E	109.5
H62B—C62—H62C	109.5	H71D—C71B—H71E	109.5
O22—C66—N16	125.2 (7)	C70B—C71B—H71F	109.5
O22—C66—H66	117.4	H71D—C71B—H71F	109.5
N16—C66—H66	117.4	H71E—C71B—H71F	109.5
N16—C67—H67A	109.5	C73B—C72B—O26B	111.0 (11)
N16—C67—H67B	109.5	C73B—C72B—H72C	109.4
H67A—C67—H67B	109.5	O26B—C72B—H72C	109.4
N16—C67—H67C	109.5	C73B—C72B—H72D	109.4
H67A—C67—H67C	109.5	O26B—C72B—H72D	109.4
H67B—C67—H67C	109.5	H72C—C72B—H72D	108.0
N16—C68—H68A	109.5	C72B—C73B—H73D	109.5
N16—C68—H68B	109.5	C72B—C73B—H73E	109.5
H68A—C68—H68B	109.5	H73D—C73B—H73E	109.5
N16—C68—H68C	109.5	C72B—C73B—H73F	109.5
H68A—C68—H68C	109.5	H73D—C73B—H73F	109.5
H68B—C68—H68C	109.5	H73E—C73B—H73F	109.5
O18—Mn1—O7	71.17 (9)		
			
O2—C1—C2—C3	−10.1 (5)	O18—Mn1—O1—N1	−104.5 (2)
N1—C1—C2—C3	169.4 (3)	O7—Mn1—O1—N1	−46.0 (3)
O2—C1—C2—C7	169.7 (3)	O13—Mn1—O1—N1	169.2 (2)
N1—C1—C2—C7	−10.8 (5)	O17—Mn1—O1—N1	58.3 (2)
C7—C2—C3—C4	0.2 (5)	O4—Mn1—O1—N1	−16.5 (2)
C1—C2—C3—C4	−180.0 (3)	O10—Mn1—O1—N1	124.5 (2)
C2—C3—C4—C5	−0.7 (6)	O18—Mn1—O1—Mn3	69.81 (15)
C3—C4—C5—C6	0.1 (6)	O7—Mn1—O1—Mn3	128.34 (16)
C4—C5—C6—C7	1.1 (7)	O13—Mn1—O1—Mn3	−16.45 (13)
C5—C6—C7—O3	179.9 (4)	O17—Mn1—O1—Mn3	−127.38 (14)
C5—C6—C7—C2	−1.6 (6)	O4—Mn1—O1—Mn3	157.78 (15)
C3—C2—C7—O3	179.3 (3)	O10—Mn1—O1—Mn3	−61.2 (2)
C1—C2—C7—O3	−0.5 (5)	N1—C1—O2—Mn3	−1.7 (4)
C3—C2—C7—C6	1.0 (5)	C2—C1—O2—Mn3	177.8 (2)
C1—C2—C7—C6	−178.9 (3)	O15—Mn3—O2—C1	−175.9 (2)
O5—C8—C9—C10	−0.4 (5)	O1—Mn3—O2—C1	4.1 (2)
N2—C8—C9—C10	−179.7 (3)	O21—Mn3—O2—C1	−87.9 (2)
O5—C8—C9—C14	−179.3 (4)	O20—Mn3—O2—C1	93.4 (2)
N2—C8—C9—C14	1.3 (6)	C6—C7—O3—Mn2	−156.9 (3)
C14—C9—C10—C11	−0.2 (6)	C2—C7—O3—Mn2	24.7 (5)
C8—C9—C10—C11	−179.2 (4)	N1—Mn2—O3—C7	−27.0 (3)
C9—C10—C11—C12	−0.8 (7)	N6—Mn2—O3—C7	162.5 (3)
C10—C11—C12—C13	1.0 (8)	O5—Mn2—O3—C7	71.3 (3)
C11—C12—C13—C14	−0.3 (8)	O16—Mn2—O3—C7	−115.0 (3)
C12—C13—C14—O6	180.0 (5)	C8—N2—O4—Mn2	−9.1 (4)
C12—C13—C14—C9	−0.7 (7)	Mn6—N2—O4—Mn2	174.45 (13)
C10—C9—C14—O6	−179.7 (4)	C8—N2—O4—Mn1	134.8 (3)
C8—C9—C14—O6	−0.8 (6)	Mn6—N2—O4—Mn1	−41.7 (2)
C10—C9—C14—C13	0.9 (6)	N1—Mn2—O4—N2	107.9 (2)
C8—C9—C14—C13	179.9 (4)	N6—Mn2—O4—N2	−81.5 (2)
O8—C15—C16—C17	−5.5 (5)	O5—Mn2—O4—N2	9.5 (2)
N3—C15—C16—C17	172.5 (3)	O16—Mn2—O4—N2	−164.0 (2)
O8—C15—C16—C21	176.9 (3)	N1—Mn2—O4—Mn1	−33.59 (14)
N3—C15—C16—C21	−5.2 (5)	N6—Mn2—O4—Mn1	137.00 (14)
C21—C16—C17—C18	1.0 (6)	O5—Mn2—O4—Mn1	−131.95 (14)
C15—C16—C17—C18	−176.8 (4)	O16—Mn2—O4—Mn1	54.47 (13)
C16—C17—C18—C19	−0.6 (6)	O18—Mn1—O4—N2	−18.3 (2)
C17—C18—C19—C20	−0.1 (7)	O7—Mn1—O4—N2	52.54 (19)
C18—C19—C20—C21	0.5 (7)	O13—Mn1—O4—N2	−98.9 (3)
C19—C20—C21—O9	−179.2 (4)	O1—Mn1—O4—N2	−111.8 (2)
C19—C20—C21—C16	−0.1 (6)	O17—Mn1—O4—N2	142.5 (2)
C17—C16—C21—O9	178.4 (4)	O10—Mn1—O4—N2	103.1 (2)
C15—C16—C21—O9	−4.0 (6)	O18—Mn1—O4—Mn2	124.15 (14)
C17—C16—C21—C20	−0.6 (5)	O7—Mn1—O4—Mn2	−165.00 (15)
C15—C16—C21—C20	177.1 (3)	O13—Mn1—O4—Mn2	43.5 (3)
O11—C22—C23—C28	175.2 (3)	O1—Mn1—O4—Mn2	30.66 (13)
N4—C22—C23—C28	−5.0 (5)	O17—Mn1—O4—Mn2	−75.09 (13)
O11—C22—C23—C24	−2.8 (5)	O10—Mn1—O4—Mn2	−114.44 (14)
N4—C22—C23—C24	177.0 (3)	N2—C8—O5—Mn2	7.1 (4)
C28—C23—C24—C25	0.0 (6)	C9—C8—O5—Mn2	−172.3 (3)
C22—C23—C24—C25	178.0 (4)	O3—Mn2—O5—C8	171.5 (2)
C23—C24—C25—C26	−1.0 (7)	O4—Mn2—O5—C8	−9.0 (2)
C24—C25—C26—C27	0.7 (8)	N1—Mn2—O5—C8	−98.3 (2)
C25—C26—C27—C28	0.7 (8)	N6—Mn2—O5—C8	82.3 (2)
C26—C27—C28—O12	179.9 (5)	O16—Mn2—O5—C8	12.7 (5)
C26—C27—C28—C23	−1.7 (7)	C13—C14—O6—Mn6	−177.0 (3)
C24—C23—C28—O12	179.6 (4)	C9—C14—O6—Mn6	3.7 (7)
C22—C23—C28—O12	1.7 (6)	N2—Mn6—O6—C14	−4.8 (4)
C24—C23—C28—C27	1.3 (6)	N12—Mn6—O6—C14	157.3 (4)
C22—C23—C28—C27	−176.6 (4)	O8—Mn6—O6—C14	−102.0 (4)
O14—C29—C30—C31	−17.5 (5)	C15—N3—O7—Mn6	1.3 (3)
N5—C29—C30—C31	160.4 (4)	Mn5—N3—O7—Mn6	174.71 (12)
O14—C29—C30—C35	161.5 (3)	C15—N3—O7—Mn1	−131.1 (2)
N5—C29—C30—C35	−20.6 (5)	Mn5—N3—O7—Mn1	42.3 (2)
C35—C30—C31—C32	1.1 (6)	N2—Mn6—O7—N3	−97.6 (2)
C29—C30—C31—C32	−179.8 (4)	N12—Mn6—O7—N3	100.4 (2)
C30—C31—C32—C33	−1.6 (7)	O8—Mn6—O7—N3	−1.1 (2)
C31—C32—C33—C34	0.6 (7)	N2—Mn6—O7—Mn1	36.35 (14)
C32—C33—C34—C35	0.9 (7)	N12—Mn6—O7—Mn1	−125.61 (13)
C33—C34—C35—O15	−178.8 (4)	O8—Mn6—O7—Mn1	132.89 (13)
C33—C34—C35—C30	−1.4 (6)	O18—Mn1—O7—N3	−178.4 (2)
C31—C30—C35—O15	177.7 (4)	O13—Mn1—O7—N3	−107.6 (2)
C29—C30—C35—O15	−1.3 (6)	O1—Mn1—O7—N3	117.4 (2)
C31—C30—C35—C34	0.3 (5)	O17—Mn1—O7—N3	8.9 (2)
C29—C30—C35—C34	−178.7 (3)	O4—Mn1—O7—N3	87.8 (2)
N6—C41—C42—N7	0.0 (5)	O10—Mn1—O7—N3	−55.9 (2)
N8—C46—C47—N9	−0.9 (5)	O18—Mn1—O7—Mn6	46.38 (12)
N10—C51—C52—N11	0.2 (5)	O13—Mn1—O7—Mn6	117.25 (12)
N12—C56—C57—N13	0.4 (5)	O1—Mn1—O7—Mn6	−17.7 (2)
O2—C1—N1—O1	−3.1 (5)	O17—Mn1—O7—Mn6	−126.28 (13)
C2—C1—N1—O1	177.4 (3)	O4—Mn1—O7—Mn6	−47.40 (12)
O2—C1—N1—Mn2	178.8 (2)	O10—Mn1—O7—Mn6	168.91 (14)
C2—C1—N1—Mn2	−0.7 (5)	N3—C15—O8—Mn6	−0.2 (4)
O3—Mn2—N1—C1	15.5 (3)	C16—C15—O8—Mn6	177.8 (3)
O4—Mn2—N1—C1	−161.9 (3)	O6—Mn6—O8—C15	−175.8 (2)
O5—Mn2—N1—C1	−83.5 (3)	O7—Mn6—O8—C15	0.7 (2)
O16—Mn2—N1—C1	112.8 (3)	N2—Mn6—O8—C15	93.6 (2)
O3—Mn2—N1—O1	−162.7 (2)	N12—Mn6—O8—C15	−85.8 (2)
O4—Mn2—N1—O1	19.9 (2)	C20—C21—O9—Mn5	−171.4 (3)
O5—Mn2—N1—O1	98.3 (2)	C16—C21—O9—Mn5	9.6 (6)
O16—Mn2—N1—O1	−65.4 (2)	N3—Mn5—O9—C21	−5.1 (3)
O5—C8—N2—O4	0.5 (5)	N8—Mn5—O9—C21	−175.9 (3)
C9—C8—N2—O4	179.8 (3)	O11—Mn5—O9—C21	89.2 (3)
O5—C8—N2—Mn6	176.1 (3)	O17—Mn5—O9—C21	−85.6 (3)
C9—C8—N2—Mn6	−4.6 (5)	C22—N4—O10—Mn5	2.3 (3)
O6—Mn6—N2—C8	5.4 (3)	Mn4—N4—O10—Mn5	−177.45 (12)
O7—Mn6—N2—C8	−172.6 (3)	C22—N4—O10—Mn1	131.5 (2)
N12—Mn6—N2—C8	−73.5 (5)	Mn4—N4—O10—Mn1	−48.2 (2)
O8—Mn6—N2—C8	108.5 (3)	N3—Mn5—O10—N4	92.5 (2)
O6—Mn6—N2—O4	−179.0 (2)	N8—Mn5—O10—N4	−96.8 (2)
O7—Mn6—N2—O4	3.1 (2)	O11—Mn5—O10—N4	−1.4 (2)
N12—Mn6—N2—O4	102.2 (4)	O17—Mn5—O10—N4	172.2 (2)
O8—Mn6—N2—O4	−75.9 (2)	N3—Mn5—O10—Mn1	−40.10 (12)
O8—C15—N3—O7	−0.7 (5)	N8—Mn5—O10—Mn1	130.54 (12)
C16—C15—N3—O7	−178.7 (3)	O11—Mn5—O10—Mn1	−134.07 (12)
O8—C15—N3—Mn5	−172.3 (2)	O17—Mn5—O10—Mn1	39.53 (9)
C16—C15—N3—Mn5	9.8 (5)	O18—Mn1—O10—N4	−21.9 (2)
O9—Mn5—N3—C15	−5.0 (3)	O7—Mn1—O10—N4	−82.4 (2)
O10—Mn5—N3—C15	172.0 (3)	O13—Mn1—O10—N4	56.59 (19)
N8—Mn5—N3—C15	59.0 (8)	O1—Mn1—O10—N4	102.9 (2)
O11—Mn5—N3—C15	−109.8 (3)	O17—Mn1—O10—N4	−176.7 (2)
O17—Mn5—N3—C15	105.2 (3)	O4—Mn1—O10—N4	−133.45 (19)
O9—Mn5—N3—O7	−176.6 (2)	O18—Mn1—O10—Mn5	111.30 (12)
O10—Mn5—N3—O7	0.4 (2)	O7—Mn1—O10—Mn5	50.83 (11)
N8—Mn5—N3—O7	−112.6 (7)	O13—Mn1—O10—Mn5	−170.19 (13)
O11—Mn5—N3—O7	78.6 (2)	O1—Mn1—O10—Mn5	−123.89 (13)
O17—Mn5—N3—O7	−66.4 (2)	O17—Mn1—O10—Mn5	−43.50 (11)
O11—C22—N4—O10	−1.9 (5)	O4—Mn1—O10—Mn5	−0.23 (17)
C23—C22—N4—O10	178.3 (3)	N4—C22—O11—Mn5	0.7 (4)
O11—C22—N4—Mn4	177.7 (2)	C23—C22—O11—Mn5	−179.5 (3)
C23—C22—N4—Mn4	−2.1 (5)	O9—Mn5—O11—C22	178.6 (2)
O12—Mn4—N4—C22	8.5 (3)	O10—Mn5—O11—C22	0.4 (2)
O13—Mn4—N4—C22	−172.0 (3)	N3—Mn5—O11—C22	−92.0 (2)
O19—Mn4—N4—C22	−84.4 (3)	N8—Mn5—O11—C22	90.0 (2)
N10—Mn4—N4—C22	99.4 (3)	O17—Mn5—O11—C22	−9.9 (3)
O12—Mn4—N4—O10	−171.8 (2)	C27—C28—O12—Mn4	−172.5 (3)
O13—Mn4—N4—O10	7.6 (2)	C23—C28—O12—Mn4	9.2 (6)
O19—Mn4—N4—O10	95.3 (2)	O14—Mn4—O12—C28	165.5 (3)
N10—Mn4—N4—O10	−81.0 (2)	N4—Mn4—O12—C28	−11.7 (3)
O14—C29—N5—O13	−3.1 (5)	O19—Mn4—O12—C28	80.6 (3)
C30—C29—N5—O13	179.1 (3)	N10—Mn4—O12—C28	−102.2 (3)
O14—C29—N5—Mn3	−176.0 (2)	C29—N5—O13—Mn4	10.3 (3)
C30—C29—N5—Mn3	6.2 (5)	Mn3—N5—O13—Mn4	−175.95 (13)
O15—Mn3—N5—C29	18.5 (3)	C29—N5—O13—Mn1	141.0 (2)
O1—Mn3—N5—C29	−161.5 (3)	Mn3—N5—O13—Mn1	−45.3 (3)
O21—Mn3—N5—C29	−69.6 (3)	O14—Mn4—O13—N5	−10.41 (19)
O20—Mn3—N5—C29	109.4 (3)	N4—Mn4—O13—N5	166.7 (2)
O15—Mn3—N5—O13	−154.2 (2)	O19—Mn4—O13—N5	74.57 (19)
O1—Mn3—N5—O13	25.8 (2)	N10—Mn4—O13—N5	−102.8 (2)
O21—Mn3—N5—O13	117.7 (2)	O14—Mn4—O13—Mn1	−143.30 (13)
O20—Mn3—N5—O13	−63.2 (2)	N4—Mn4—O13—Mn1	33.85 (13)
N7—C40—N6—C41	−0.6 (5)	O19—Mn4—O13—Mn1	−58.32 (12)
N7—C40—N6—Mn2	−168.5 (3)	N10—Mn4—O13—Mn1	124.28 (13)
C42—C41—N6—C40	0.4 (5)	O18—Mn1—O13—N5	−59.8 (2)
C42—C41—N6—Mn2	168.7 (3)	O7—Mn1—O13—N5	−123.33 (19)
O3—Mn2—N6—C40	−172.3 (3)	O1—Mn1—O13—N5	34.06 (19)
O4—Mn2—N6—C40	5.2 (3)	O17—Mn1—O13—N5	132.40 (19)
O5—Mn2—N6—C40	−73.2 (3)	O4—Mn1—O13—N5	21.3 (3)
O16—Mn2—N6—C40	90.3 (3)	O10—Mn1—O13—N5	−175.6 (2)
O3—Mn2—N6—C41	21.9 (3)	O18—Mn1—O13—Mn4	69.93 (12)
O4—Mn2—N6—C41	−160.7 (3)	O7—Mn1—O13—Mn4	6.37 (17)
O5—Mn2—N6—C41	120.9 (3)	O1—Mn1—O13—Mn4	163.76 (13)
O16—Mn2—N6—C41	−75.5 (3)	O17—Mn1—O13—Mn4	−97.90 (12)
N6—C40—N7—C42	0.6 (5)	O4—Mn1—O13—Mn4	150.97 (16)
N6—C40—N7—C43	179.2 (4)	O10—Mn1—O13—Mn4	−45.91 (11)
C41—C42—N7—C40	−0.3 (5)	N5—C29—O14—Mn4	−5.9 (4)
C41—C42—N7—C43	−178.9 (4)	C30—C29—O14—Mn4	171.9 (3)
C44—C43—N7—C40	−115.0 (5)	O12—Mn4—O14—C29	−171.6 (2)
C44—C43—N7—C42	63.3 (6)	O13—Mn4—O14—C29	9.0 (2)
N9—C45—N8—C46	−0.6 (4)	O19—Mn4—O14—C29	−79.2 (2)
N9—C45—N8—Mn5	−178.3 (2)	N10—Mn4—O14—C29	97.3 (2)
C47—C46—N8—C45	1.0 (5)	C34—C35—O15—Mn3	−145.4 (3)
C47—C46—N8—Mn5	178.6 (3)	C30—C35—O15—Mn3	37.3 (5)
O9—Mn5—N8—C45	−11.8 (3)	O2—Mn3—O15—C35	142.7 (3)
O10—Mn5—N8—C45	170.9 (3)	N5—Mn3—O15—C35	−39.5 (3)
N3—Mn5—N8—C45	−75.9 (8)	O21—Mn3—O15—C35	53.3 (3)
O11—Mn5—N8—C45	92.9 (3)	O20—Mn3—O15—C35	−128.9 (3)
O17—Mn5—N8—C45	−121.5 (3)	O17—C36—O16—Mn2	−69.2 (4)
O9—Mn5—N8—C46	171.0 (3)	C37—C36—O16—Mn2	109.6 (4)
O10—Mn5—N8—C46	−6.3 (3)	O3—Mn2—O16—C36	−159.1 (3)
N3—Mn5—N8—C46	106.9 (7)	O4—Mn2—O16—C36	21.2 (3)
O11—Mn5—N8—C46	−84.3 (3)	N1—Mn2—O16—C36	112.3 (3)
O17—Mn5—N8—C46	61.3 (3)	N6—Mn2—O16—C36	−71.0 (3)
N8—C45—N9—C47	0.0 (4)	O5—Mn2—O16—C36	−0.2 (5)
N8—C45—N9—C48	−178.5 (4)	O16—C36—O17—Mn1	28.4 (5)
C46—C47—N9—C45	0.5 (5)	C37—C36—O17—Mn1	−150.5 (3)
C46—C47—N9—C48	179.1 (4)	O16—C36—O17—Mn5	168.0 (2)
C49—C48—N9—C45	84.9 (6)	C37—C36—O17—Mn5	−10.9 (5)
C49—C48—N9—C47	−93.4 (7)	O18—Mn1—O17—C36	91.3 (4)
N11—C50—N10—C51	0.0 (5)	O7—Mn1—O17—C36	109.4 (3)
N11—C50—N10—Mn4	173.7 (2)	O13—Mn1—O17—C36	−121.3 (3)
C52—C51—N10—C50	−0.1 (5)	O1—Mn1—O17—C36	−39.6 (3)
C52—C51—N10—Mn4	−173.4 (3)	O4—Mn1—O17—C36	33.9 (3)
O12—Mn4—N10—C50	25.9 (3)	O10—Mn1—O17—C36	−177.1 (3)
O13—Mn4—N10—C50	−156.6 (3)	O18—Mn1—O17—Mn5	−60.1 (3)
O14—Mn4—N10—C50	121.3 (3)	O7—Mn1—O17—Mn5	−42.05 (9)
N4—Mn4—N10—C50	−63.4 (3)	O13—Mn1—O17—Mn5	87.31 (10)
O12—Mn4—N10—C51	−162.1 (4)	O1—Mn1—O17—Mn5	168.97 (8)
O13—Mn4—N10—C51	15.5 (4)	O4—Mn1—O17—Mn5	−117.57 (9)
O14—Mn4—N10—C51	−66.7 (4)	O10—Mn1—O17—Mn5	31.44 (8)
N4—Mn4—N10—C51	108.6 (4)	O9—Mn5—O17—C36	−3.4 (3)
N10—C50—N11—C52	0.1 (5)	O10—Mn5—O17—C36	174.4 (3)
N10—C50—N11—C53	−179.5 (4)	N3—Mn5—O17—C36	−88.5 (3)
C51—C52—N11—C50	−0.2 (5)	N8—Mn5—O17—C36	84.1 (3)
C51—C52—N11—C53	179.5 (4)	O11—Mn5—O17—C36	−174.7 (3)
C54—C53—N11—C50	7.8 (7)	O9—Mn5—O17—Mn1	144.91 (10)
C54—C53—N11—C52	−171.7 (5)	O10—Mn5—O17—Mn1	−37.27 (9)
N13—C55—N12—C56	−0.5 (5)	N3—Mn5—O17—Mn1	59.80 (11)
N13—C55—N12—Mn6	173.6 (3)	N8—Mn5—O17—Mn1	−127.52 (11)
C57—C56—N12—C55	0.0 (5)	O11—Mn5—O17—Mn1	−26.3 (2)
C57—C56—N12—Mn6	−174.0 (3)	O19—C38—O18—Mn1	−4.8 (5)
O6—Mn6—N12—C55	−167.2 (3)	C39—C38—O18—Mn1	177.2 (3)
O7—Mn6—N12—C55	11.9 (3)	O7—Mn1—O18—C38	94.9 (3)
N2—Mn6—N12—C55	−88.1 (5)	O13—Mn1—O18—C38	−36.2 (3)
O8—Mn6—N12—C55	90.0 (3)	O1—Mn1—O18—C38	−113.7 (3)
O6—Mn6—N12—C56	5.7 (3)	O17—Mn1—O18—C38	114.0 (3)
O7—Mn6—N12—C56	−175.2 (3)	O4—Mn1—O18—C38	169.7 (3)
N2—Mn6—N12—C56	84.8 (5)	O10—Mn1—O18—C38	33.0 (3)
O8—Mn6—N12—C56	−97.1 (3)	O18—C38—O19—Mn4	24.7 (6)
N12—C55—N13—C57	0.7 (5)	C39—C38—O19—Mn4	−157.2 (3)
N12—C55—N13—C58	−172.8 (4)	O12—Mn4—O19—C38	−171.1 (4)
C56—C57—N13—C55	−0.7 (5)	O13—Mn4—O19—C38	11.4 (4)
C56—C57—N13—C58	172.9 (4)	O14—Mn4—O19—C38	93.8 (4)
C59—C58—N13—C55	86.8 (6)	N4—Mn4—O19—C38	−81.7 (4)
C59—C58—N13—C57	−85.5 (6)	N14—C60—O20—Mn3	−177.5 (3)
O20—C60—N14—C61	0.6 (7)	O15—Mn3—O20—C60	170.4 (3)
O20—C60—N14—C62	178.9 (4)	O1—Mn3—O20—C60	−10.2 (3)
O22—C66—N16—C67	−4.0 (11)	O2—Mn3—O20—C60	−92.2 (3)
O22—C66—N16—C68	179.6 (7)	N5—Mn3—O20—C60	80.3 (3)
C1—N1—O1—Mn3	6.3 (3)	O15—Mn3—O21—C63	−178.8 (4)
Mn2—N1—O1—Mn3	−175.27 (12)	O1—Mn3—O21—C63	1.9 (4)
C1—N1—O1—Mn1	−178.9 (2)	O2—Mn3—O21—C63	83.8 (4)
Mn2—N1—O1—Mn1	−0.5 (3)	N5—Mn3—O21—C63	−88.7 (4)
O2—Mn3—O1—N1	−5.57 (19)	Mn3—O21—C63—N15	178.0 (3)
N5—Mn3—O1—N1	176.7 (2)	O21—C63—N15—C64	0.4 (6)
O21—Mn3—O1—N1	83.8 (2)	O21—C63—N15—C65	176.6 (4)
O20—Mn3—O1—N1	−94.0 (2)	C72—O26—C70—C71	−171.7 (17)
O2—Mn3—O1—Mn1	179.45 (15)	C70—O26—C72—C73	−74 (2)
N5—Mn3—O1—Mn1	1.69 (15)	C72B—O26B—C70B—C71B	169 (2)
O21—Mn3—O1—Mn1	−91.16 (14)	C70B—O26B—C72B—C73B	177 (2)
O20—Mn3—O1—Mn1	91.03 (14)		

### Hydrogen-bond geometry (Å, °)

**Table:**

*D*—H···*A*	*D*—H	H···*A*	*D*···*A*	*D*—H···*A*
O24—H24A···O22	0.84	2.09	2.876 (7)	156.
O24B—H24B···O19	0.84	2.15	2.98 (2)	170.
O25—H25A···O24B	0.84 (2)	2.35 (2)	3.05 (4)	140 (6)
O25—H25B···O22	0.84 (2)	1.99 (2)	2.776 (17)	156 (4)
